# Anatomy of Subterranean Organs of Medicinally Used Cardueae and Related Species and its Value for Discrimination

**DOI:** 10.3797/scipharm.1010-05

**Published:** 2010-12-02

**Authors:** Elisabeth Fritz, Johannes Saukel

**Affiliations:** Department of Pharmacognosy, University of Vienna, Althanstrasse 14, 1090, Vienna, Austria

**Keywords:** Root anatomy, Asteraceae, Microscopy, Plant anatomy

## Abstract

Numerous species of the Asteraceae, the composites, are famous for their use in both traditional and conventional medicine. Reliable anatomical descriptions of these plants and of possible adulterations provide a basis for fast identification and cheap purity controls of respective medicinal drugs by means of light microscopy. Nevertheless, detailed comparative studies on root and rhizome anatomy of valuable as well as related inconsiderable composite plants are largely missing yet. The presented study aims to narrow this gap by performing anatomical analyses of roots and rhizomes of 16 species belonging to the tribe Cardueae, of formerly and currently used drugs as well as their near relatives as potential adulterations (*Carlina acaulis* L., *Carlina vulgaris* L., *Arctium lappa* L., *Arctium tomentosum* Mill., *Carduus defloratus* L., *Carduus personata* (L.) Jacq, *Cirsium arvense* (L.) Scop., *Cirsium vulgare* (Savi) Ten., *Cirsium erisithales* (Jacq.) Scop., *Onopordum acanthium* L., *Silybum marianum* (L.) Gaertn., *Rhaponticum scariosum* Lam., *Centaurea jacea* L., *Centaurea scabiosa* L., *Centaurea cyanus* L.*, Cnicus benedictus* L.). A detailed verbal and graphical survey of the analysed anatomical features is provided. Several characters were finally extracted which allow for discrimination of the examined species and may be effectively used for drug quality controls.

## Introduction

**Fig. 1.**
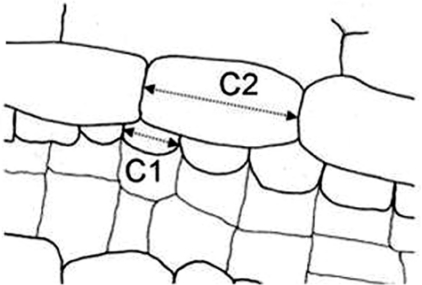
Secretory duct in longitudinal section build from two adjacent layers of cells differing in length. The quotient C1:C2 is used to discriminate between three types of secretory ducts (fig. cited: [12]).

The Asteraceae represent one of the largest plant families comprising at least 23,000 species and about 1,600 genera [1]. Numerous composites such as *Carlina acaulis, Arctium lappa* and *Taraxacum officinale,* to mention only a few, have a long history in both traditional and conventional medicine. Light microscopy is a common and effective method for the identification of pharmaceutically useful plants and their adulterations. Hence, detailed knowledge of the anatomy of the diverse plant parts used as drugs is required for quality control. Yet, reliable comparative studies of the anatomy of subterranean organs of medicinally exploited Asteraceae species and their close relatives are rare. Most studies date back to about 1900 and concentrate on the secretory system of the family tribes Cardueae (characterized by resin ducts) and Cichorieae (laticifers) in particular [2–5]. Most work, however, concerned the aerial vegetative organs and did not aim to discriminate species. Some recent studies deal with the subterranean organ anatomy of diverse Asteraceae species [e.g. 6–11]. However, the investigated species were of no medicinal value and not of a Western European origin. An additional recent study concentrates on the secretory structures of the subterranean organs of various species of the tribe Cardueae and demonstrated - based on five identified types of secretory ducts - the value of anatomical characters for the discrimination of composite plants [12]:

- Secretory ducts of type SD1 and SD2a / SD2b are of schizogenous origin and distinguished based on the C1:C2-quotient (i.e. the length of the cells lining the ducts [C1] divided by the length of adjacent cells [C2], longitudinal section, see fig. 1 [fig cited: 12]).
- Secretory ducts of type SD3 show cell lysis: the large lumen is filled with the remnants of the cells.
- Secretory ducts of type SD4 seem to be actually intercellular space, filled with diverse substances secreted by the adjacent parenchyma cells.

Although the secretory system proofed valuable for species discrimination, the anatomy of entire roots and rhizomes is still insufficiently described. So far a single study only focuses on medicinal drugs [13] by comparing the root anatomy of *Taraxacum* spp., *Leontodon* sp., *Aposeris foetida,* and *Hypochaeris* sp..

In order to narrow this gap of knowledge, a comprehensive study on the anatomy of the subterranean organs of 32 genera and 57 species from the tribes *Cardueae* (22 species) and *Cichorieae* (35 species) used in medicine or occurring as potential adulterations was initiated. The anatomy of these plants was analysed in detail and a database of typical anatomical features created [14].

The present work deals with the tribe Cardueae and analyses the underground part anatomy of species possessing a taproot or dominating rhizome. Taxa with a fibrous root system were excluded. The focus thereby lies on formerly or currently medicinally used species of the Cardueae, which have been used for medical purposes since long:

Thus already Hieronymus Bock [15] mentions *Carlina acaulis* L. as a plant highly valued by people as a diaphoretic and diuretic medicine and against worms. The roots soaked in vinegar were also known as appropriate remedy against scabies and tetter [16]. Today, this plant is still used in traditional medicine and serves as an ingredient of various liquids such as Swedish bitters elixir, as a diuretic as well as a stomachic remedy and against skin diseases [17, 18].

Even though rarely used nowadays, another species of the genus *Carlina, Carlina vulgaris* L., still has a place in traditional medicine. The species is applied against nocturnal enuresis and to cure frightened babies [19].

The medicinally use of *Arctium lappa* L. reaches back to the ancient times. Dioskurides [20] praises the effects of its roots as an expectorans (the antitussive activity was confirmed by Kardošová [21]) and its application on luxations and sprains. Lonicerus [22] mentions its effects against asthma. The usage of *A. lappa* oil as hair restorer was first mentioned in 1673 [Pankovius, 1673 in: 23] and is still popular among people. In traditional medicine, *A. lappa* – along with *Arctium tomentosum* Mill. – is still valued as a plant with antirheumatic, diuretic and diaphoretic effects [17, 19]. Recent studies document a gastroprotective activity [24] and hepatoprotective effects [25].

Although officinal in former times (Radix et Herba Spinae albae seu Cardui tomentosi; [23]), these days, *Onopordum acanthium* is no longer used except in anthroposophical medicine (Onopordi acanthi herba) [17]. The roots (together with the sap of the fresh leaves) were reported to be useful against stiff neck and opisthotonus [26, 20] and as a diaphoretic and laxative remedy [23].

*Rhaponticum* has been used in Chinese, Tibetan and Mongol medicine for more than 5,000 years [27]. *Rhaponticum carthamoides* (Willd.) Iljin. was studied currently as it is supposed to possess various positive effects on memory, blood, cardiovascular and nervous system and on physiological functions such as work capacity and sexual function (reviewed by Kokoska [28]). Łotocka & Geszprych [27] dealt with the anatomy and secretory structures of *R. carthamoides* but the identification of the plant and its roots remained problematic as comparative material from other species of the genus is missing.

On that account the anatomy of *Rhaponticum scariosum* Lam. – the only species of the genus native to Austria (the area mainly addressed here) – has been included in our studies. Though *R. scariosum* has been used in former times as substitute to *Rheum palmatum* [29] it is not applied any longer.

Carduus defloratus L., Carduus personata (L.) Jacq, Cirsium arvense (L.) Scop., Cirsium vulgare (Savi) Ten., Cirsium erisithales (Jacq.) Scop. Silybum marianum L., Centaurea jacea L., Centaurea scabiosa L., Centaurea cyanus and Cnicus benedictus L. have been examined as near relatives of the taxa mentioned above.

## Results

Reliable anatomical root and rhizome characters discriminating between the various species investigated in this study were identified. These features are proofed valuable for the characterisation of the drugs and may be used for quality control:

The following main anatomical features of roots and rhizomes can be used for purity and quality control in pharmacy: the overall distribution and proportions of the principal tissues in transverse section, the fine structure of the cork, vessel types, the occurrence of fibers including their maximum diameter and wall structure, and, finally, the occurrence of sclereids and secretory ducts in diverse tissues.

Our studies demonstrated that even within one single plant the diameter of the largest vessels may differ largely depending on their position along the root axis. In transverse sections the dimension of the largest vessels of *Silybum marianum*, for instance, varied largely with their position along the taproot: the maximum diameter decreased from 245 μm near the tip of the root to 113 μm 1.5 cm below the hypocotyle. However, in other samples of the same species this parameter varied insignificantly only.

The example of *S. marianum* points out that the diameter of vessels, though a popular feature used for quality control of drugs, has to be carefully considered as preparations of drugs usually are available as a cut formulation. Therefore, standardisation of the analysed root parts appears impossible.

For the purpose of standardization measured values given in the following microscopical descriptions always refer to a section of the root axis just below the hypocotyle (see Experimental). As the occurrence of secretory ducts is of particular importance for the taxonomic identification of the species described in the following, respective data have been included from [12].

### Microscopical descriptions and discriminative anatomical characters

#### Carlina acaulis L. (fig. 2)

Secondary root: cork thin-walled; cortex durable or lost in course of rhytidome formation; endodermal resin ducts are in size a multiple of the diameter of the surrounding parenchyma cells, lost together with the cortex; secondary phloem dominant, comparable in extension to the vascular cylinder, with fibers arranged in bundles, rays with crystalline needles; secondary xylem consists of fibers and vessels; vessels reticulate and simple, strongly bordered, up to 114 μm in diameter; medullary rays multiseriate, parenchymatous, with crystalline needles of the same type occurring in the phloem; secretory ducts of type **SD1** and **SD2a** (average quotient **C1:C2** <0,4 [12]), size a multiple of the diameter of the surrounding parenchyma cells, located in the medullary rays and in the phloem rays; pith missing; sclereids missing;

#### Carlina vulgaris L. (fig. 3)

Secondary root: cork thin-walled sometimes with crystalloids; cortex enduring; distinct endodermis with endodermal resin ducts – usually up to 10 surrounding cells at maximum; secondary phloem broad, but of lesser radial extension than the vascular cylinder, with secretory ducts of type **SD2** (C1:C2 >0,48); secondary xylem dominated by fibers, few vessels dispersed over the transverse section, strongly bordered, up to 59 μm in diameter; medullary rays biseriate; pith missing; sclereids, laticifers missing; fibers in secondary phloem missing;

#### Arctium lappa L. and A. tomentosum Mill. (fig. 4)

The following description applies to both examined species:

Secondary root: broad phellem thin-walled – cortex lost in course of rhytidome formation; endodermal resin ducts lost together with the cortex; secondary phloem broad, but of lesser radial extension than the vascular cylinder, possibly with fibers arranged in bundles; secondary xylem mostly with vessels in rows – in the center single-rowed, in the outer part multiple-rowed, often combined with fibers, mainly reticulate, also weakly bordered up to 89 μm (*A. lappa*) / 125 μm (*A. tomentosum*) in diameter; medullary rays multiseriate, unlignified; cells of medullary rays often obliterating and easily ripping apart; pith missing; sclereids, crystalloids missing; secretory ducts besides endodermal resin ducts missing;

The early loss of the cortex with its endodermal resin ducts deprives the genus *Arctium* of a character otherwise valuable for taxonomic comparisons with taxa outside of the Cardueae.

#### Onopordum acanthium L. (fig. 5)

Secondary root: cork thin-walled, sometimes with crystalloids; small cortex enduring; distinct endodermis with endodermal resin ducts usually up to 6 surrounding cells at maximum; secondary phloem usually broader than cortex, but of lesser radial extension than the vascular cylinder, with fibers single and arranged in bundles; secondary xylem with fibers alternating with parenchymatous cells, vessels dispersed, strongly bordered, up to 150 μm in diameter; multiseriate medullary rays multiseriate; pith missing; sclereids missing; secretory ducts beside endodermal resin ducts missing;

The root anatomy of *Silybum marianum* resembles that of *O. acanthium*. A distinction among these species may be possible with proceeding secondary growth: rhytidome formation within *S. marianum* versus maintenance of a durable cortex with well visible endodermal resin ducts in *O. acanthium*. The root anatomy of *Cnicus benedictus* and *O. acanthium* is similar. The diameter of the vessels may serve as the only discriminative character available (*C. benedictus*: <100 μm, *O. acanthium* up to 150 μm) with the value of this feature questionable

#### Rhaponticum scariosum Lam. (fig. 6)

Secondary root: cork thin-walled; cortex enduring; endodermal resin ducts; secondary phloem dominant, often almost comparable in extension to the vascular cylinder, with fibers arranged in bundles possible, secretory ducts type **SD2** located in fascicular position between the phloem rays, circularly arranged, forming a triangle; secondary xylem with bundles of fibers in conjunction with vessels, more or less circularly arranged relative to the centre of the xylem, vessels reticulate, up to 120 μm in diameter, with secretory ducts type **SD2** located in fascicular position in the secondary xylem between the medullary rays; medullary rays broad, multiseriate, unlignified; pith missing; sclereids, crystalloids missing;

The anatomy of *R. scariosum* clearly differs from all other species examined in this study based on the arrangement of the secretory ducts. Although the roots develop ducts both within the secondary phloem and the secondary xylem like *Carlina acaulis*, in *R. scariosum* ducts are found in fascicular position, i.e. between the medullary rays. In contrast, ducts occur within the medullary in all other taxa examined.

### Carduus sp. and Cirsium sp

*Carduus defloratus*, *C. personata*, *Cirsium arvense*, *C. vulgare* and *C. erisithales* are close relatives of the medicinally used species described above. These species are representatives of the subtribe Carduinae (just like *Onopordum acanthium* and *Arctium* spp.) and studied given their potential occurrence as adulterations.

**Fig. 7.**
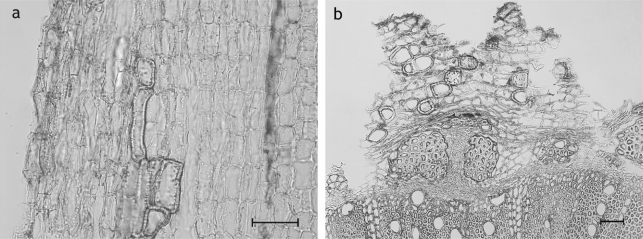
*Cirsium arvense* root: sclereids (as an important discriminative character) developed within the cortex; a: longitudinal section; b: transverse sections; scale bars are 50 μm

**Fig. 8.**
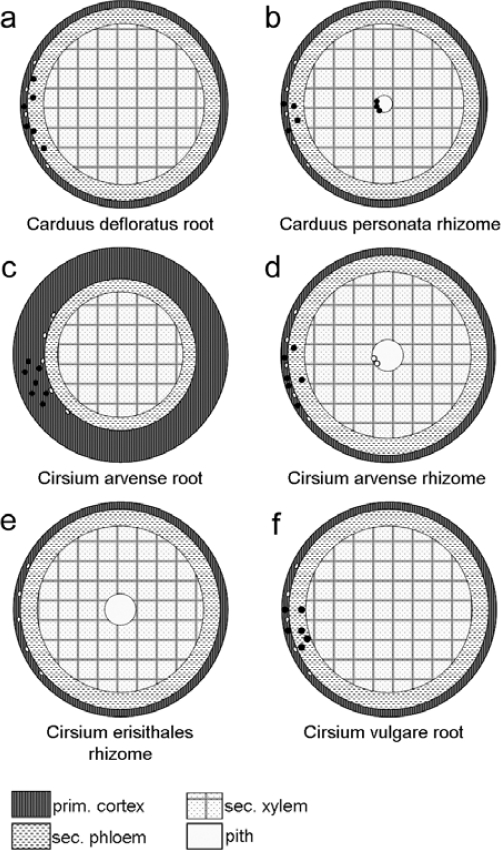
Schematic view of *Carduus* sp. and *Cirsium* sp. (transverse section): small ellipses mark the position of the enodermal resin ducts (endoSDs) of the cortex and the ducts of type SD2, black points represent the sclereids *a: Carduus defloratus*: Secondary root: sclereids in cortex and sec. phloem, endoSDs lost together with the cortex in course of rhytidome formation; SDs besides endoSDs missing; *b: Carduus personata*: Rhizome with secondary growth: sclereids in cortex, secondary phloem and as transition between vascular bundles and pith; endoSDs or remnants of them visible; SDs besides endoSDs missing; *c,d: Cirsium arvense*: allorhizous: taproot with long part of rhizome; stoloniferous plant – spreading building rhizomes; Secondary root (c): sclereids in cortex (large intercellular spaces – aerenchyma) and secondary phloem; endoSDs regularly arranged; SDs besides endoSDs missing; Rhizome with secondary growth (d): sclereids in cortex and secondary phloem; endoSDs lost lost together with the suberizing of the cortex; SDs at the border between vascular bundle and pith; *e: Cirsium erisithales*: Rhizome with secondary growth: endoSDs lost together with the cortex due to rhytidome formation; sclereids missing; SDs besides the endoSDs missing; *f: Cirsium vulgare*: Secondary root: sclereids in cortex and secondary phloem; endoSDs lost together with the cortex due to rhytidome formation; SDs beside endoSDs missing;

The characters of highest value for the discrimination of *Carduus* and *Cirsium* (except *Cirsium erisithales*) from their medicinally used relatives is the exhibition of sclereids (fig. 7). *Cirsium erisithales*, can be distinguished instead based on its rhizome, which replaces the root developed in the other studied species (fig. 8).

#### Centaurea sp

The three species of the genus *Centaurea* investigated in this study – *C. jacea*, *C. cyanus* and *C. scabiosa* – can be well distinguished from each other based on secretory structures [12]:

*Centaurea jacea* is characterised by sclereids regularly appearing in conjunction with intercellular spaces filled by various substances secreted by the surrounding cells.

In *C. scabiosa,* large secretory ducts with lysigenous development, beside others, can be observed in the secondary phloem in fascicular position. Both mentioned secretory ducts are missing in *C. cyanus*.

## Discussion

The diameter of vessels is a parameter frequently used for the identification of medicinal drugs [e.g. 13]. Our studies, however, demonstrated that this character may largely vary, aside from the age and development of a root, even within one plant. Therefore, the root part taken for respective analyses should be standardized. This appears highly important, even so most studies did not define the exact spatial localisation and developmental stage of the analysed root sample [13]. In case of cut roots as common with commercially traded drugs, the use of not-standardized values may be misleading. Furthermore, varying preparation methods may influence the dimension of the vessels.

**Fig. 9.**
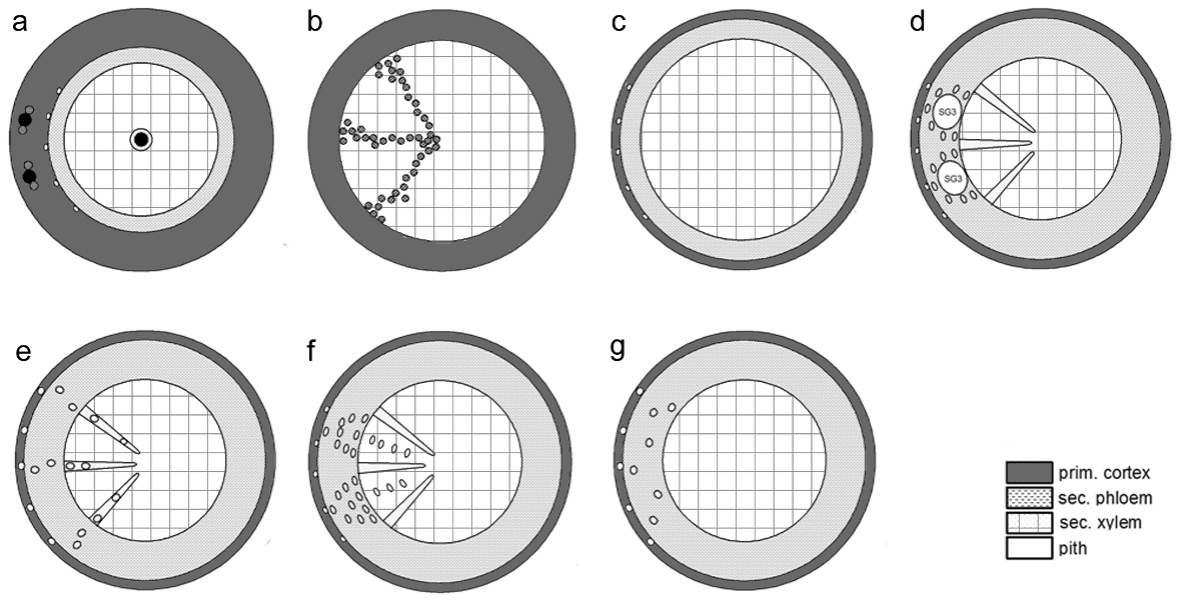
Schematic view of the various roots studied: small ellipses mark the position of the endodermal resin ducts of the cortex and the ducts of type SD2; a: black points represent the sclereids in conjunction with secretory ducts (grey dots), b: points represent the vessels (b); a: *Centaurea jacea*; b: *Arctium lappa, A. tomentosum*; c: *Onopordum acanthium, Silybum marianum, Cnicus benedictus*; d: *Centaurea scabiosa*; e: *Carlina acaulis*; f: *Rhaponticum scariosum*; g: *Carlina vulgaris, Centaurea cyanus*;

**Fig. 10.**
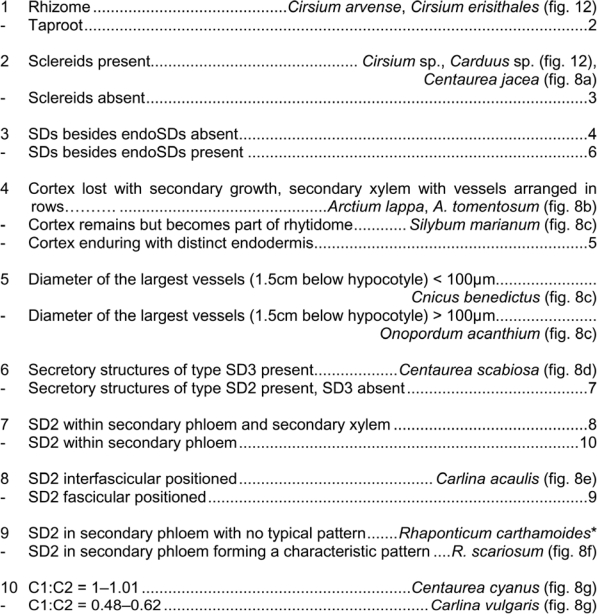
Key of the subterranean organs of the species examined in this study; * Data concerning *R. carthamoides* is based on Łotocka & Geszprych [27]

Discrimination between most of the examined species proofed possible based on the observed diversity and differentiation of the various anatomical features analysed. A summary of the root anatomy as seen in transverse sections is presented for these species in figures 9 and 10. This overview is intended to provide a taxonomic key.

Strikingly, all species of the genera *Cirsium and Carduus* as well as *Centaurea jacea* can be easily identified in medicinal drugs by the unique possession of rhizomes or sclereids. The other taxa can be distinguished based on the presence or absence, type (according to [12]) and spatial location of secretory structures.

Interestingly, the anatomy of several species may vary widely within a single genus (*Centaurea* sp., *Carlina* sp.) or, in contrast, be indifferent and of no discriminative value as in the case of the genus *Arctium*. In addition, species of different genera may be quite similar in root anatomy (*Onopordum acanthium, Cnicus benedictus, Silybum marianum*).

The discrimination between the two examined representatives of the genus *Carlina*, *C. acaulis* and *C. vulgaris*, is easily possible based on anatomical features: The endodermal resin ducts of the enduring cortex of *C. vulgaris*, and *C. acaulis*. are clearly distinct in size. In conjunction with the endodermis – usually visible even in roots showing secondary growth – this feature well characterizes these species. The **C1:C2** quotient of the epithelial cells of the secretory ducts occurring in the secondary phloem as well as their spatial position provide other good discriminative features. The domination of fibers over few vessels in the secondary xylem, vessel diameter (up to 60 μm; about half the value observed in *C. acaulis*), and biseriate medullary rays with ducts missing are additional characters distinguishing the medically inconsiderable species from its valuable relative. Finally, the absence of fibers in the secondary phloem and of crystalline needles may be used for differentiation.

*Centaurea cyanus*, *C. jacea* and *C. scabiosa* differed from each other in secretory duct type (fig. 8).

According to Łotocka & Geszprych [27] an anatomical differentiation between *R. carthamoides* and *R. scariosum* seems possible. The conspicuous arrangement of the secretory ducts within the secondary phloem, arranged in regular circles around the centre thereby forming together with the phloem rays a kind of triangular pattern, however,, is not described in that study. *Rhaponticum scariosum* appears to develop a much more extensive secondary phloem than the medicinally applied species.

This study on the anatomy of several roots and rhizomes of the Cardueae reveal considerable among-species variation. Important features of discriminative value are the arrangement of principal tissues as observed in transverse section of roots and rhizomes, the tissue-dependent occurrence of fibers and sclereids as well as of various types of secretory ducts. The different duct types [12] provide a particularly valuable character for taxonomic discrimination if the type of surrounding tissue and their position relative to prominent anatomical elements such as vascular bundles is taken into account. Certainly, the arrangement and diameter of vessels and the appearance of medullary rays in the secondary xylem must not be forgotten. However, the largest diameter of vessels though can be only considered a reliable taxonomically informative character if applied to root parts standardized in terms of developmental age and spatial position.

In summary, an identification of most investigated taxa used in medicine proofed possible based on various extracted anatomical characters. Further studies into the root anatomy using a broader and more diverse taxonomic sample have to verify the feasibility of microscopic techniques for the identification of medicinally used genera and species of the Asteraceae.

## Experimental

The plant material comprised of 16 Cardueae species naturally occurring in Austria. Species were chosen respectively to their use in medicine (*Carlina acaulis*, *Carlina vulgaris*, *Arctium lappa, Arctium tomentosum, Onopordum acanthium,*) and their role as possible adulterations (*Rhaponticum scariosum, Carduus defloratus*, *Carduus personata*, *Cirsium arvense*, *Cirsium vulgare*, *Cirsium erisithales, Silybum marianum*, *Centaurea jacea*, *Centaurea scabiosa*, *Centaurea cyanus* and *Cnicus benedictus*.). To guarantee full development of the roots, the plants were collected during or following antheses. A list of the studied species including the collection history is provided in Table 1 (taxonomy follows Fischer et al., 2008 [31]). Vouchers available for all studied species are deposited in the herbarium of the Department of Pharmacognosy, University of Vienna (**WUP**). The plant material was taxonomically determined using floristic treatments covering the sampled geographic areas [31–33].

In order to include modificative effects of the environment (e.g. influence of the soil) on root anatomy, each species was collected from different locations if possible.

Anatomical analysis: The roots were examined by means of light microscopy. For preparation, a traditional method of our department was used: After boiling in water for about 10 minutes to soften the tissues, the roots were embedded in 96% ethanol for dehydration. Transverse and longitudinal sections were obtained by free hand sectioning about 1.5 cm below the hypocotyle as this position proofed to be the furthest developed region, thus providing the most information about the anatomy. The resulting sections were embedded in few drops of a solution of chloral hydrate (60% in water) and examined using a Nikon Optiphot–2 light microscope equipped with a Samsung Digimax V50 Digital Camera.

## Supporting Information



## Figures and Tables

**Fig. 2.**
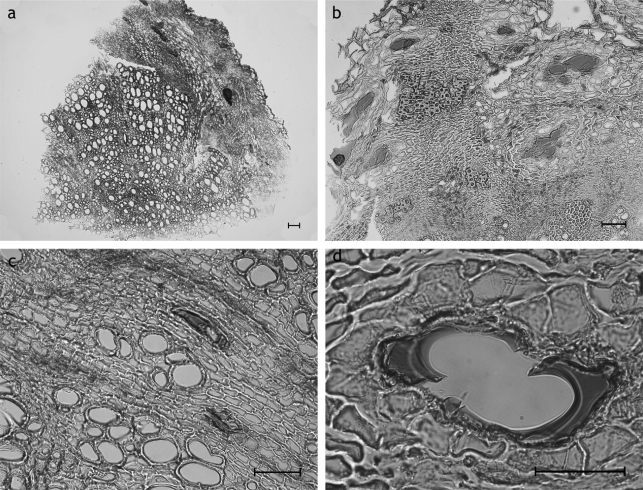
*Carlina acaulis* root: a: Overview showing the extension and arrangement of the participating tissues: the secondary xylem is the most prominent component followed in extension by the secondary phloem; cortex conspicuously small or lost in course of rhytidome formation; small cortex with endodermal resin ducts, secondary phloem and xylem with secretory ducts type SD1 within the medullary rays; b: secondary phloem having an expansion a multiple of the diameter of the surrounding parenchyma cells and with fibers arranged in bundles; c: SD1 and crystalline needles in medullary rays of the vascular cylinder; d: secretory ducts type SD1 of secondary phloem; a–d: transverse sections; scale bars are 50 μm

**Fig. 3.**
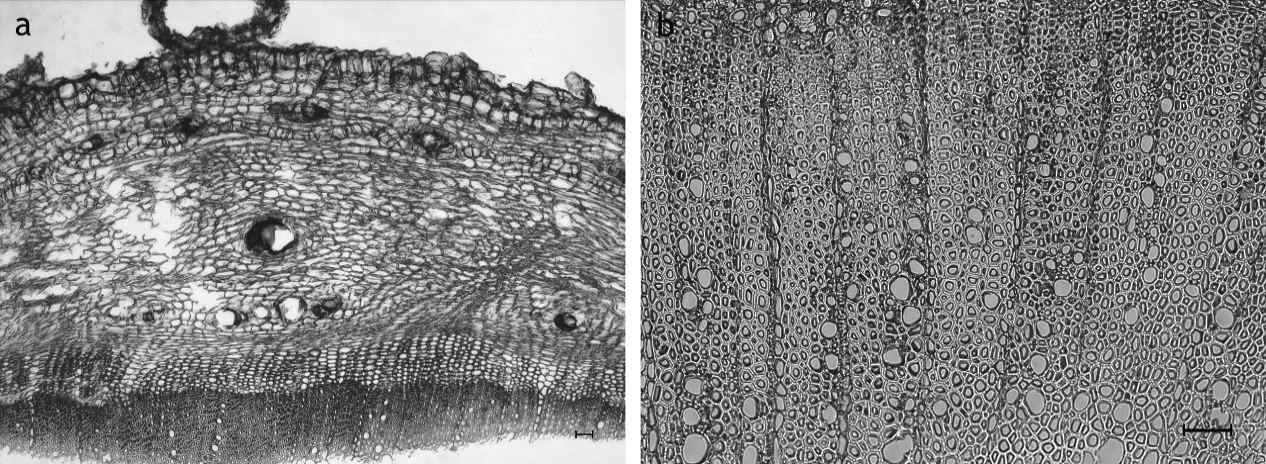
*Carlina vulgaris* root: a: overview showing distinct endodermal resin ducts and secretory ducts of the type SD2b in the secondary phloem; b: xylem dominated by fibers and with dispersed vessels, biseriate medullary rays in regular arrangement; a, b: transverse sections; scale bars are 50 μm

**Fig. 4.**
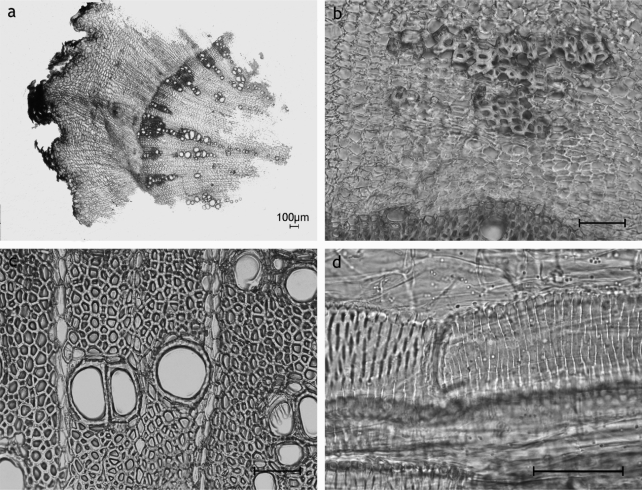
*Arctium lappa* root: a: Overview demonstrating the extension and arrangement of tissues: the secondary xylem is the most expanded component followed in extension by the secondary phloem; cortex lost in course of rhytidome formation; xylem dominated by unlignified parenchymatous cells, vessels arranged in rows; b: secondary phloem with sclereids and fibers arranged in bundles ; c: xylem with fibers and with dispersed vessels, medullary rays in regular arrangement; d: reticulate vessels; a–c: transverse sections; d: longitudinal section; scale bars are 50 μm

**Fig. 5.**
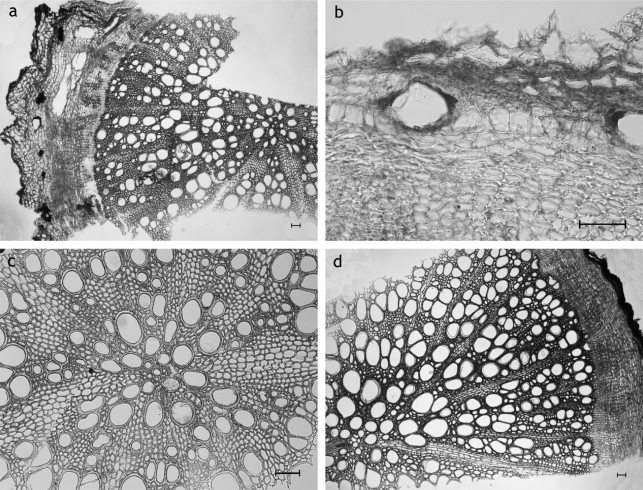
*Onopordum acanthium* root: a: Overview showing the extension and arrangement of tissues: the secondary xylem is the most expanded component followed in extension by the secondary phloem; cortex enduring with endodermal resin ducts; vascular cylinder with multiseriate medullary rays (from left to right); b: Distinct endodermis with endodermal resin ducts usually up to 6 surrounding cells at maximum; c Vascular cylinder with multiseriate medullary rays, numerous dispersed vessels, fibers; d: *Silybum marianum* root: cortex lost, secondary phloem broader than cortex but of far lesser radial extension than the vascular cylinder; a–d: transverse sections; scale bars are 50 μm

**Fig. 6.**
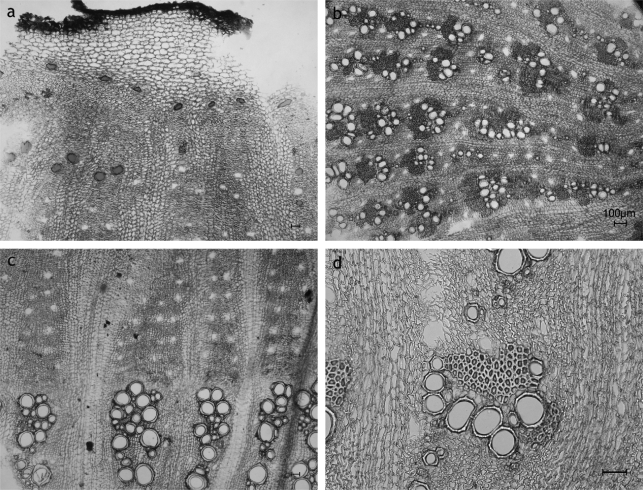
*Rhaponticum scariosum* root: a: enduring cortex with endodermal resin ducts, SD2 in secondary phloem between phloem rays; b: overview showing the extension of the vascular cylinder with vessels in groups more or less circularly arranged relative to the centre of the xylem, multiseriate medullary rays; c: secretory ducts type SD2 arranged in fascicular position between phloem rays of the secondary phloem and the medullary rays of the secondary xylem; d: group of vessels and fibers of the secondary xylem, secretory ducts SD2; a-d: transverse sections; scale bars are 50 μm

**Tab. 1.** List of species examined in this study (accessions are taxonomically arranged following the current systematic concept provided in [31]); AUT: Austria, GER: Germany, ITA: Italy, POL: Poland, SLK: Slovakia, LIE: Liechtenstein; Plant material collected by Elisabeth Fritz (EF), Christoph Dobeš (CD), Silvia Fialova (SF), Werner Lahner (WL), Günther Stadler (GS)

**Genus**	**Species**	**Location of collection**
*Carlina*	*C. acaulis* L.	AUT, Vienna, EF GER, Baden-Württemberg, Schwäbische Alb, Hohenack, Nr. 652 (WUP) Samples of a commercially traded product (Kottas Pharma, sample number 1881, 1882)
	C. vulgaris L.	POL, Gutkowo / Olsztyn, EF

*Arctium*	*A. lappa* L.	AUT, Vienna, Donauinsel, EF
	*A. tomentosum* Mill.	-, Lower Austria, Traiskirchen, EF

*Carduus*	*C. defloratus* L.	-, Lower Austria, Gippel, CD
		-, Lower Austria, Araburg, EF
	*C. personata* (L.) Jacq	-, Styria, Schneealpe, EF

*Cirsium*	*C. arvense* (L.) Scop.	SLK, Modra, Tochova Chata, SF
		AUT, Vienna, EF
	*C. vulgare* (Savi) Ten.	POL, Gutkowo / Olsztyn, EF
	*C. erisithales* (Jacq.) Scop.	AUT, Styria, Schneealpe, EF

*Onopordum*	*O. acanthium* L.	AUT, Lower Austria, Buchberg, WL
		ITA, Southern Tyrol, Vinschgau, CD

*Silybum*	*S. marianum* (L.)	AUT, Lower Austria, Buchberg, EL
	Gaertn.	SLK, Bratislava, Botanical Garden, SF

*Rhaponticum R. scariosum* Lam.		LIE, GS

*Centaurea*	*C. jacea* L.	Austria, Karnabrunn, CD
		-, Vienna, EF
	*C. scabiosa* L.	-, Vienna, JS
		Poland, Gutkowo, Olsztyn, EF
		Switzerland, Graubünden, Lavin, CD
	*C. cyanus* L. (= *Cyanus segetum* Hill., Fischer *et al.)*	Austria, Vienna, EF
		Germany, Baden-Württemberg, Kronau, CD
		Poland, Mazury, Zabie, EF

*Cnicus*	*C. benedictus* L.	Botanical Garden of the Department of
		Pharmacognosy, University of Vienna

## Supporting Information

Detailed descriptions and pictures of all species mentioned in the text are available in the online version (Format: PDF, Size: ca. 5.3 MB): http://dx.doi.org/10.3797/scipharm.1010-05.
