# Supplementary material for: Anatomy of Subterranean Organs of Medicinally Used Cardueae and Related Species and its Value for Discrimination
Source: Sci Pharm. 2010 Dec 2;79(1):157–74. doi: 10.3797/scipharm.1010-05 (PMC3097509; doi:10.3797/scipharm.1010-05)
Supplement: Supplementary file 1 [file Scipharm_2011_79_157_supporting_information.pdf]

Scientia Pharmaceutica www.scipharm.at

Open Access

Supporting Information to

Anatomy of Subterranean Organs of Medicinally Used Cardueae and Related Species and its Value for Discrimination

Elisabeth Fritz, Johannes Saukel

Published in Sci Pharm. 2011; 79: 157–174 doi:10.3797/scipharm.1010-05

Available from: http://dx.doi.org/10.3797/scipharm.1010-05

© Fritz and Saukel; licensee Österreichische Apotheker-Verlagsgesellschaft m. b. H., Vienna, Austria.

This is an Open Access article distributed under the terms of the Creative Commons Attribution License (http://creativecommons.org/licenses/by/3.0/), which permits unrestricted use, distribution, and reproduction in any medium, provided the original work is properly cited.

Table of Contents

Microscopical descriptions / pictures of

1. Carduus defloratus L.
2. Carduus personata (L.) Jacq.
3. Cirsium arvense (L.) Scop.
4. Cirsium erisithales (Jacq.) Scop.
5. Cirsium vulgare (Savi) Ten.
6. Silybum marianum L.
7. Centaurea jacea L.
8. Centaurea scabiosa L.
9. Centaurea cyanus L.
10. Cnicus benedictus L.

Scale bars are 50 µm

1. *Carduus defloratus*

secondary root: phellem thin-walled, broad – cortex lost in course of rhytidome formation, parts remain with sclereids: in transverse section small lumen, in longitudinal section resembling the adjacent parenchyma cells in size and shape; endoSDs lost together with the cortex; sec. phloem broad, but of lesser radial extension than the vascular cylinder, suberizing, with sclereids of the same type occurring in the cortex and fibers single or arranged in bundles; sec. xylem dominated by fibers often arranged in tangential bands alternating with parenchymatous cells; vessels circularly arranged, mainly reticulate, also weak bordered, up to 91 µm in diameter; medullary rays multiseriate, unlignified; pith missing; crystalloids, laticifers missing; secretory ducts besides endoSDs missing;

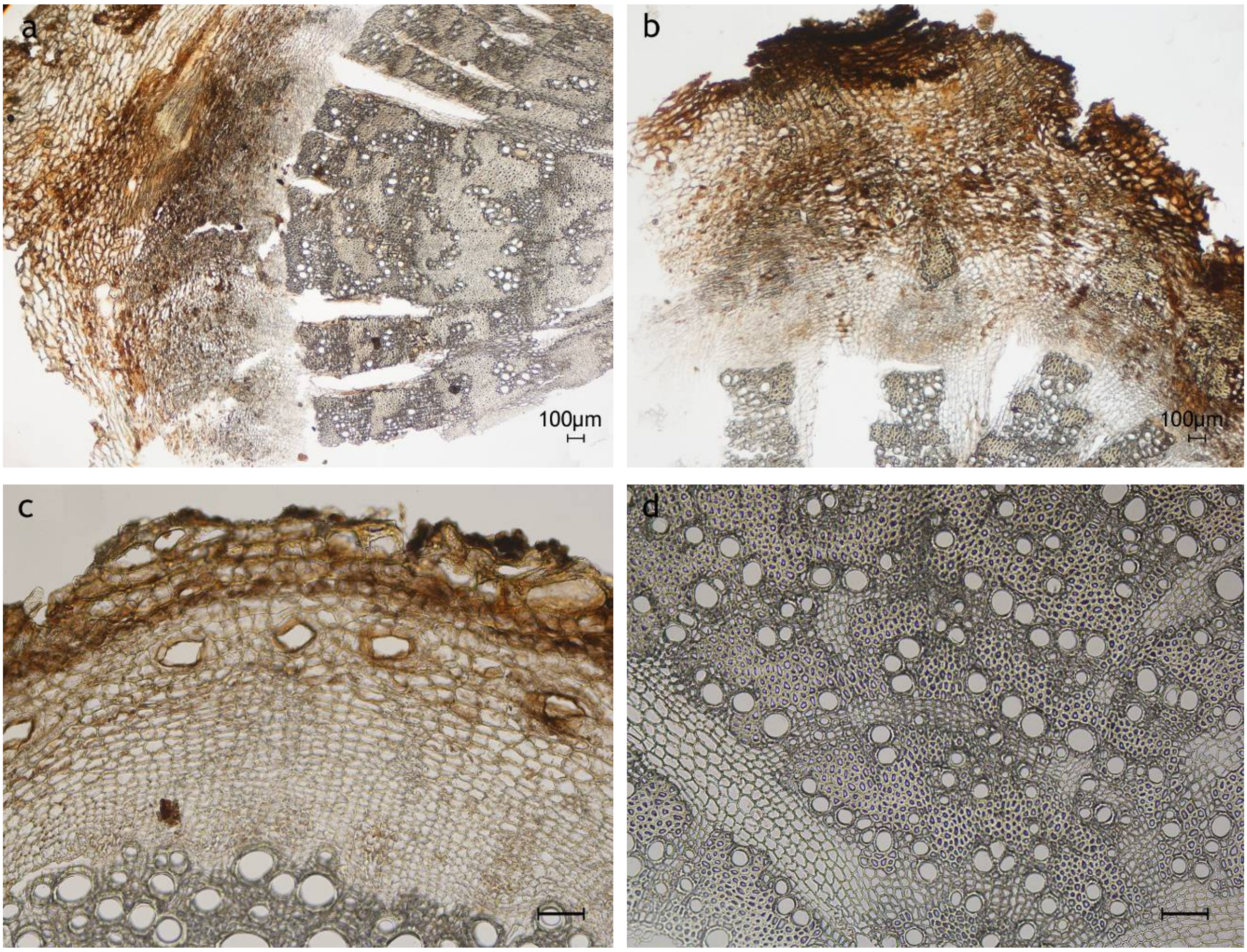

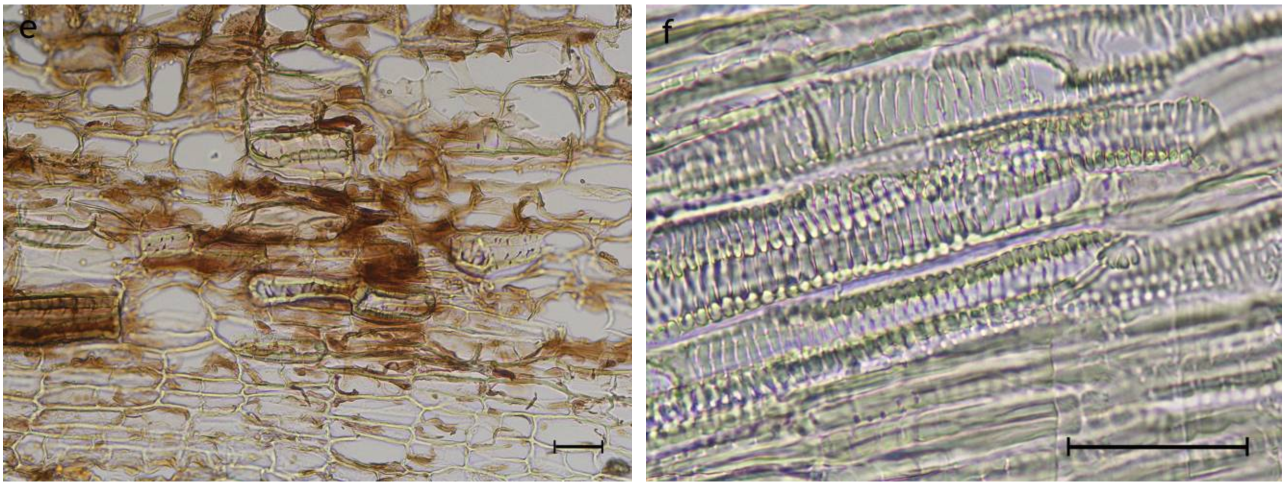

Carduus defloratus root: a: Overview showing the extension and arrangement of tissues: the secondary xylem is the most expanded component followed in extension by the secondary phloem; cortex and parts of the secondary phloem suberized; b: overview showing secondary phloem with fibers arranged in groups, bark; c: cortex with regularly arranged endoSDs of a young Carduus crassifolius root; d: vascular cylinder with vessels circularly arranged, fibers in tangential bands alternating with parenchymatous cells, medullary rays multiseriate; e: sclereids in bark resembling the adjacent parenchyma cells in size and shape; f: reticulate vessels; a–d: transverse sections; e,f: longitudinal sections;

2. *Carduus personata*

rhizome with secondary growth: thin-walled phellem; cortex suberized with sclereids either round and with small lumen or resembling the adjacent parenchyma cells in size and shape; endoSDs or remnants of them visible; secondary phloem usually broader than cortex, but of lesser radial extension than the vascular cylinder, within older rhizomes partly suberized, with sclereids of the same type occurring in the cortex and fibers single or arranged in bundles; secondary xylem with fibers dominating, vessels dispersed, reticulate and strongly bordered, up to 91 µm in diameter; sclereids as transition between vascular bundles and pith; medullary rays multiseriate with sclerenchymatous thickening; pith of thick-wanded, pitted parenchymatous cells; crystalloids, laticifers missing; secretory ducts besides endoSDs missing;

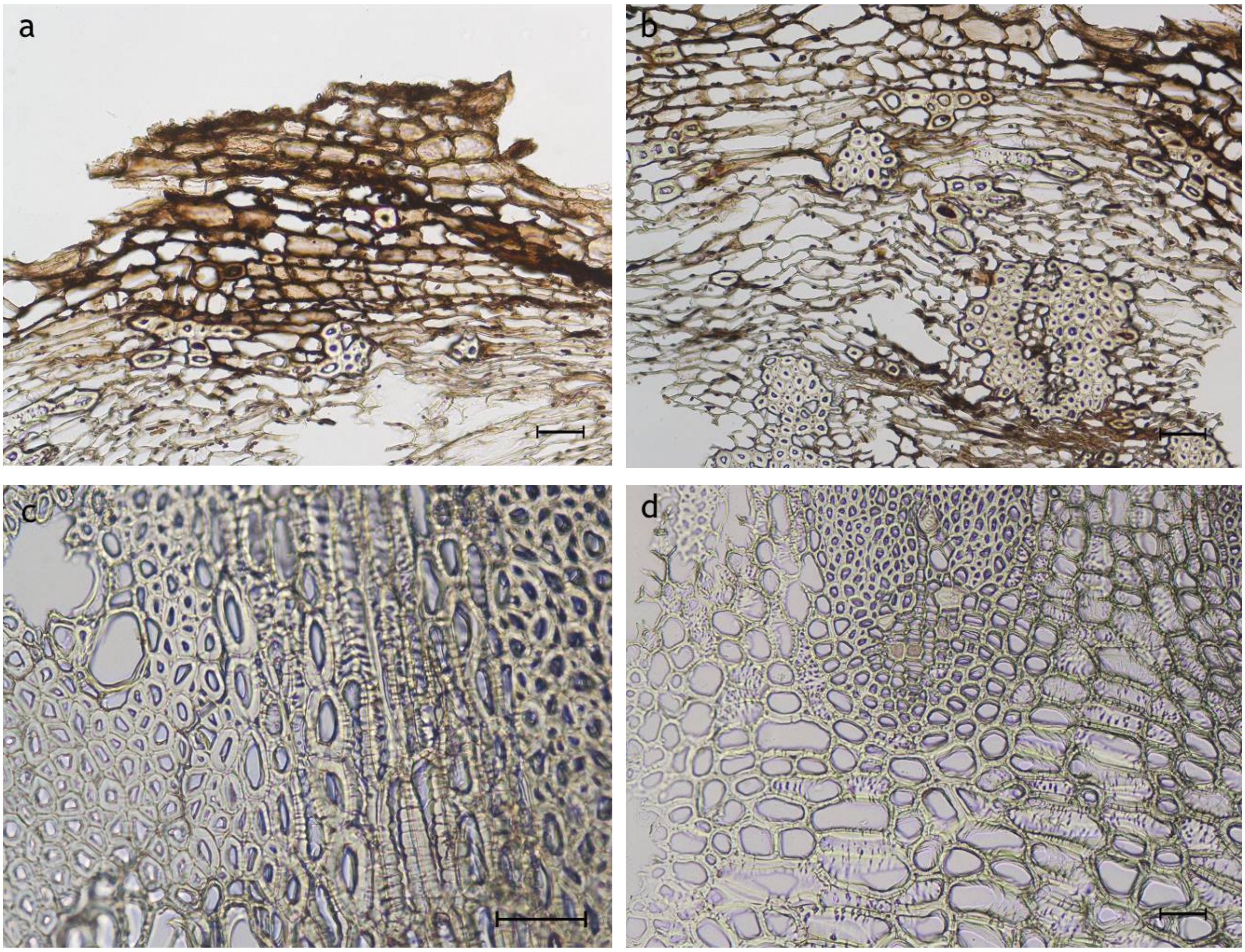

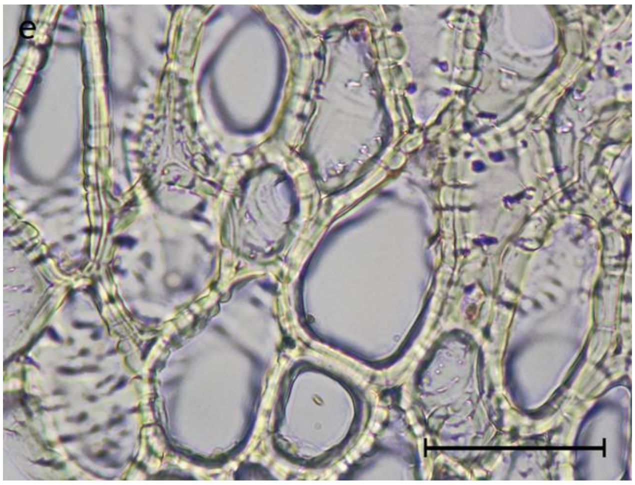

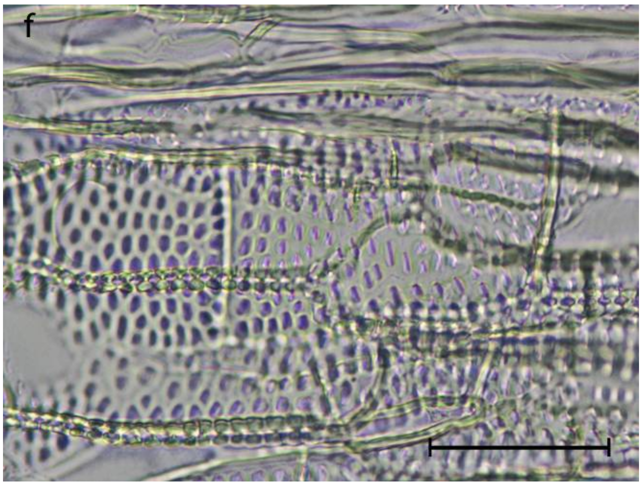

Carduus personata rhizome: a: Sclereids within the suberized cortex and secondary phloem; b: sclereids and fibers single and arranged in bundles of the secondary phloem; c: cells of medullary ray with sclerenchymatous thickening; d: vascular bundle with sclereidal cells as transition to the pith; e: pith with cells with pitted and thickened walls; f: strongly bordered vessels; a–e: transverse sections; f: longitudinal section;

3. *Cirsium arvense*

Root:

secondary root: Rhizodermis or cork thin-walled; cortex with large intercellular spaces – aerenchyma, with sclereids resembling the adjacent parenchyma cells in size and shape, large lumen; endoSDs regularly arranged; secondary phloem usually smaller than cortex, with sclereids of the same type occurring in the cortex and possibly single fibers; vascular cylinder dominating; secondary xylem with fibers, vessels strongly bordered, up to 120µm in diameter; medullary rays multiseriate; pith missing; crystalloids missing; secretory ducts besides endoSDs missing;

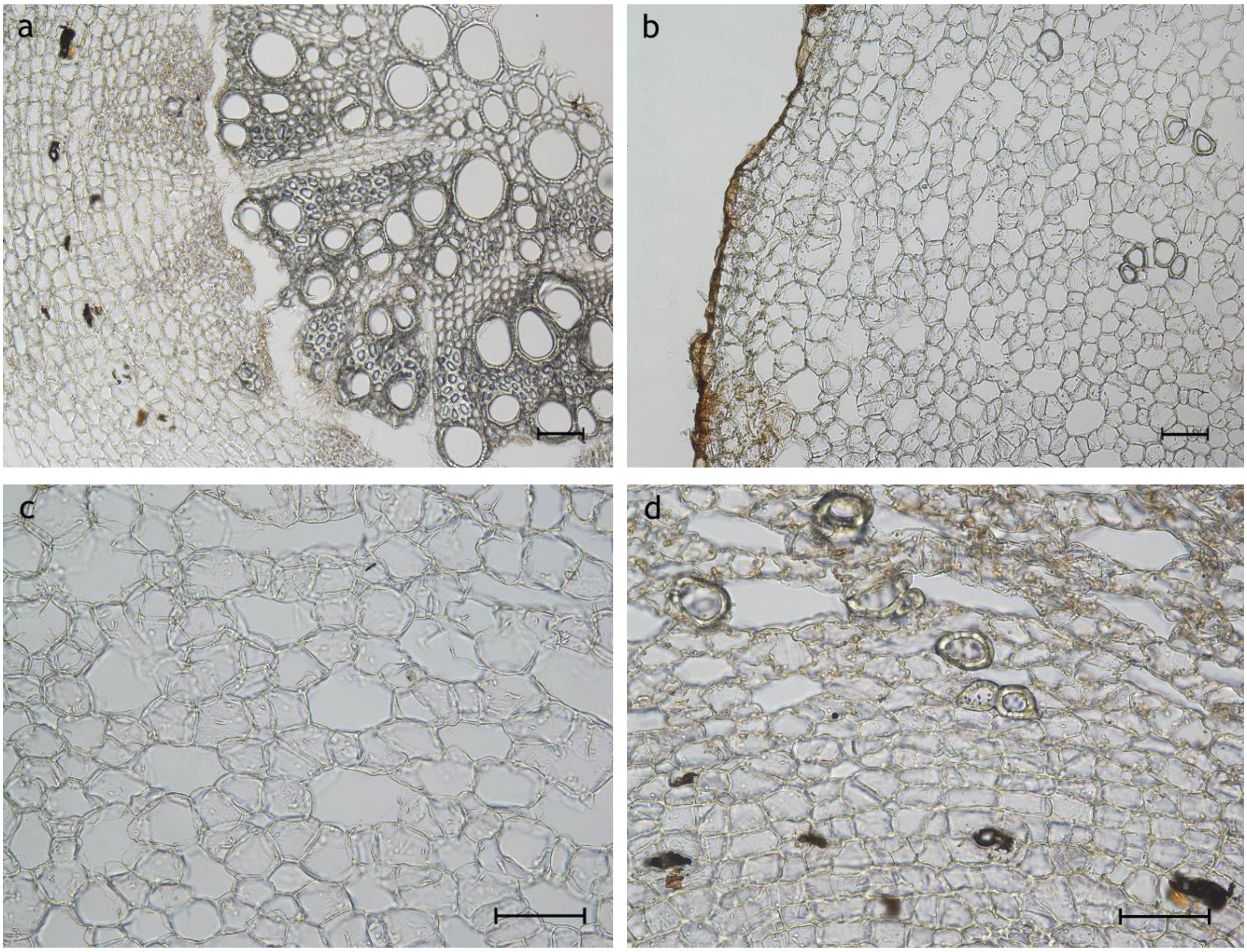

Cirsium arvense root: a: Overview showing the extension and arrangement of the vascular cylinder: the secondary xylem is the most expanded component followed in extension by the cortex; regularly arranged endoSDs; b: cortex with sclereids resembling the adjacent parenchyma cells in size and shape, large lumen; c: aerenchyma of the cortex; d: sclereids and endoSDs of the cortex; a–d: transverse sections;

Rhizome:

rhizome with secondary growth: phellem thin-walled; cortex suberizing with sclereids resembling the adjacent parenchyma cells in size and shape, large lumen, tissue frequently ruptured; endoSDs lost lost together with the suberizing of the cortex; secondary phloem conspicuously small with sclereids of the same type occurring in the cortex, fibers building a cap over the vascular bundle; wide radial extension of the dominating vascular cylinder; secondary xylem dominated by fibers, vessels dispersed, strongly bordered, up to 79 µm in diameter; secretory ducts at the border between vascular bundle and pith; medullary rays multiseriate; pith of cells with slightly thickened, pitted cell wall; crystalloids, laticifers missing;

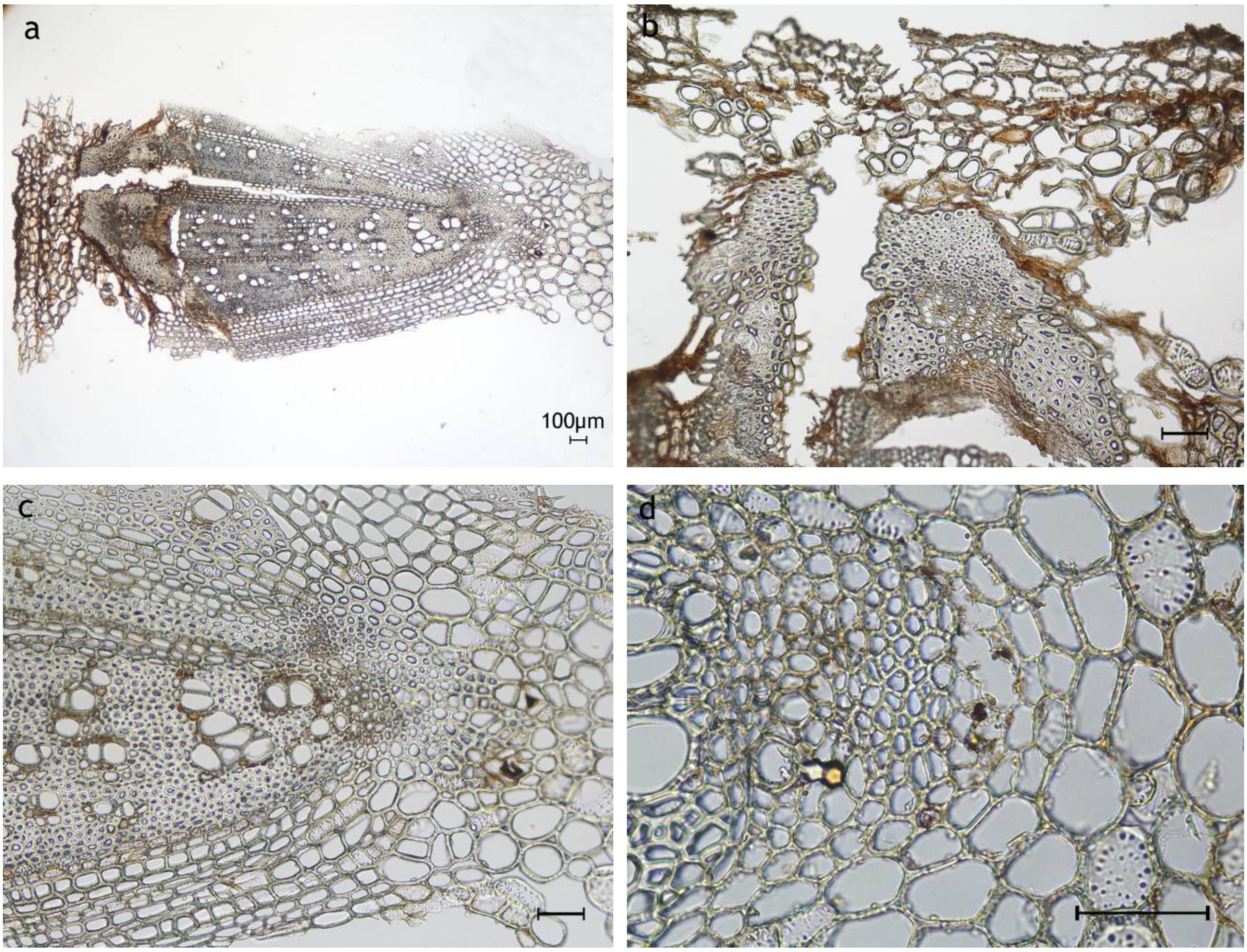

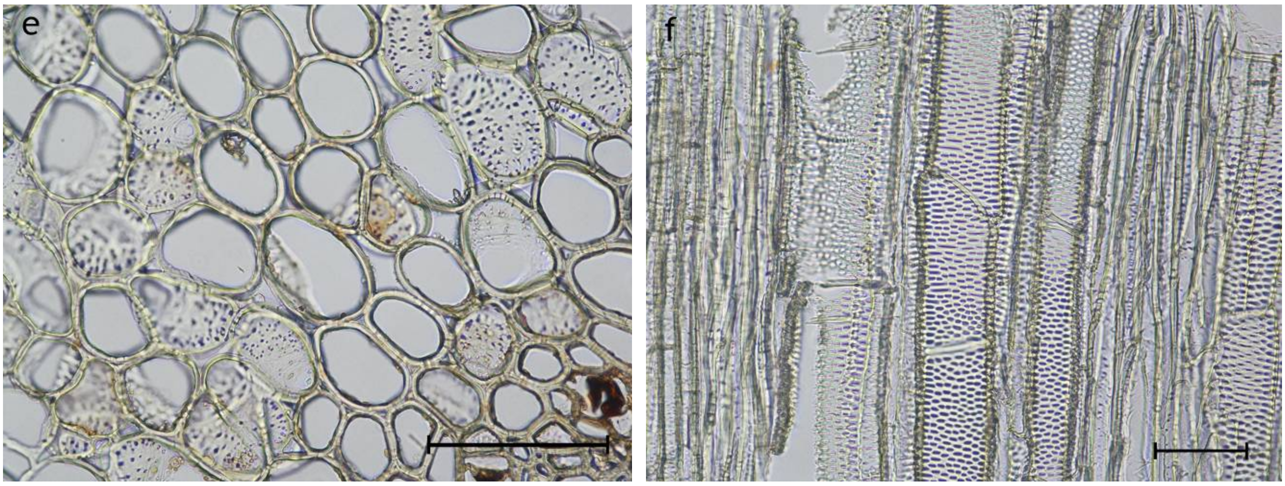

Cirsium arvense rhizome: a: Overview showing the extension and arrangement of tissues: the secondary xylem is the most expanded component, cortex and secondary phloem conspicuously small, suberizing; b: sclereids resembling the adjacent parenchyma cells in size and shape within the suberized cortex, secondary phloem with fibers building a cap over the vascular bundle; c: overview of the transition between vascular bundle and pith; d: secretory ducts at the border between vascular bundle and pith; e: pith with slightly thickened cell walls; f: strongly bordered vessels; a-e: transverse sections; f: longitudinal section;

4. *Cirsium erisithales*

rhizome with secondary growth: phellem thin-walled; cortex lost in course of rhytidome formation; endoSDs lost together with the cortex; secondary phloem broad, but of lesser radial extension than the vascular cylinder, with fibers in groups arranged in circular bands in fascicular position above the vascular bundles alternating with bands of parenchymatous cells; secondary xylem dominated by fibers, vessels circularly arranged, reticulate, mainly strongly bordered, up to 102 µm in diameter; medullary rays multiseriate; pith of thin-walled cells; crystalloids missing; sclereids, laticifers, crystalloids missing; further secretory ducts besides the endoSDs missing;

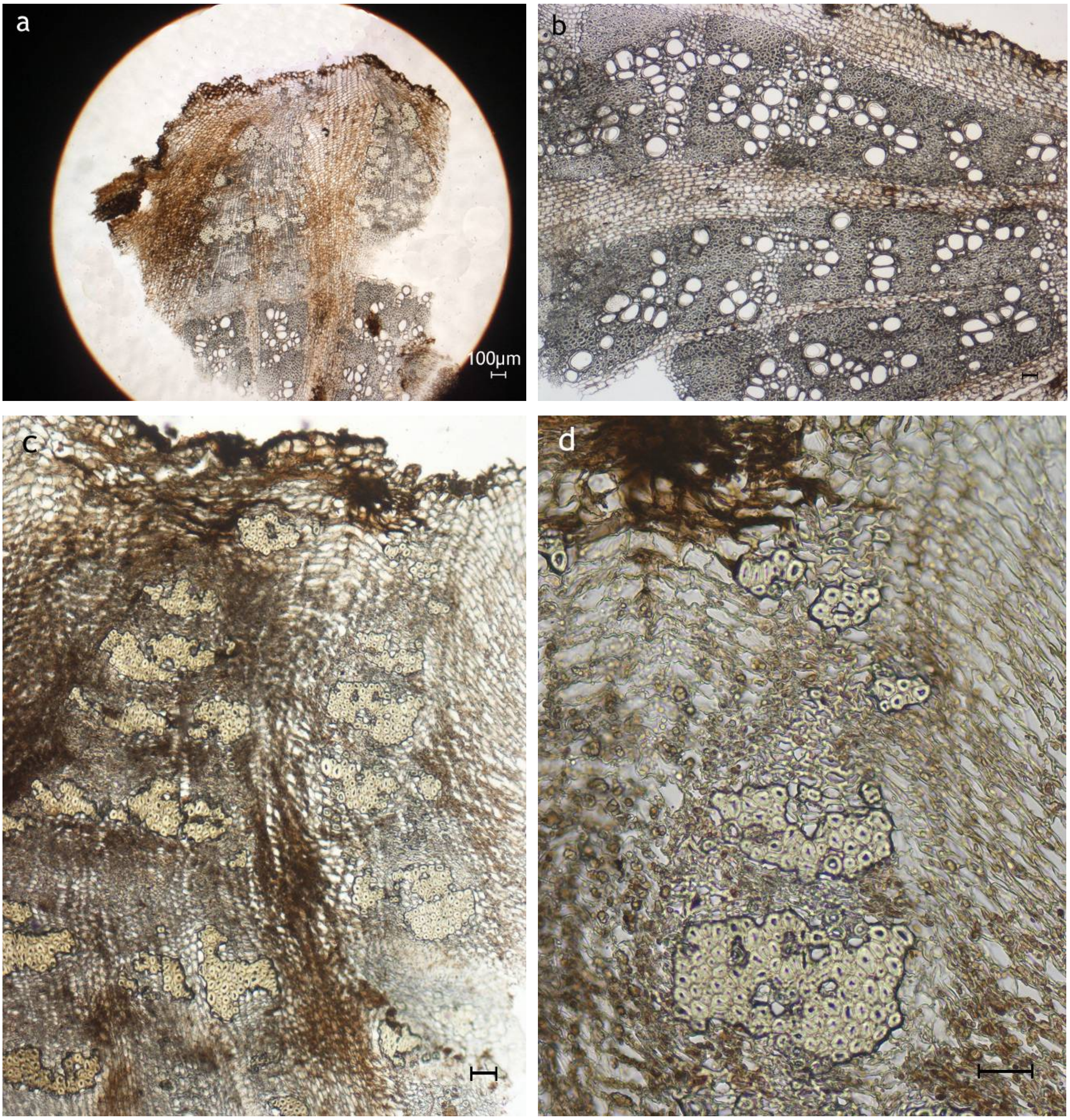

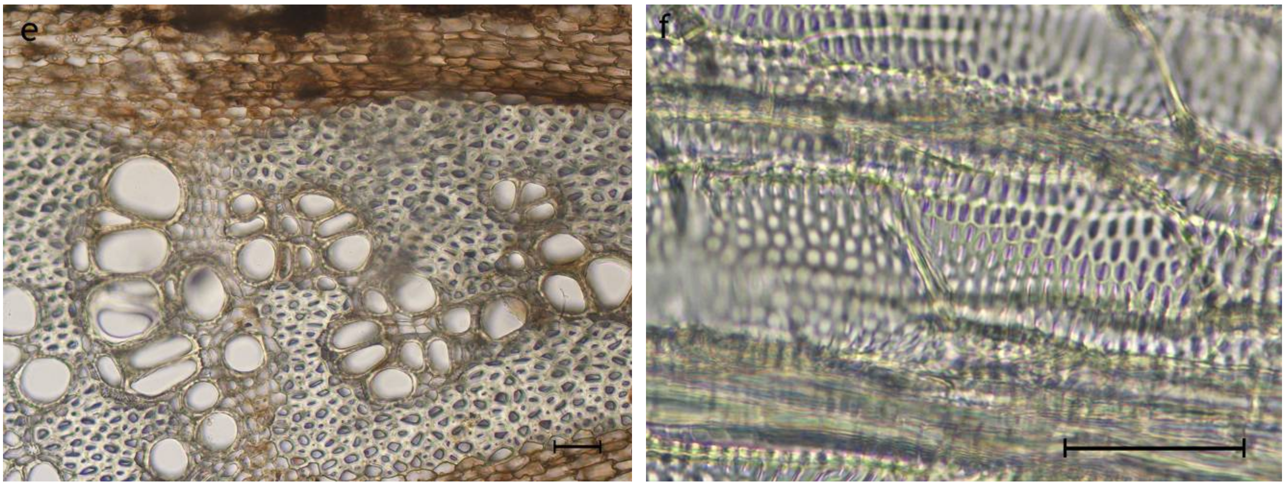

Cirsium erisithales rhizome: a: Overview showing the extension and arrangement of tissues: cortex lost in course of rhytidome formation, secondary phloem broad, but of lesser radial extension than the vascular cylinder; b: vascular cylinder dominated by fibers, vessels circularly arranged, medullary rays multiseriate; c: bundles of fibers in arranged in circular bands within the secondary phloem; d: fibers of the secondary phloem; e: vessels between alternating bands of fibers and parenchymatous cells; f: strongly bordered vessels; a–e: transverse sections; f: longitudinal sections

5. *Cirsium vulgare*

secondary root: phellem thin-walled; cortex lost, with sclereids resembling the adjacent parenchyma cells in size and shape, large lumen; endoSDs lost together with the cortex; secondary phloem broader than cortex, but of lesser radial extension than the vascular cylinder, with sclereids of the same type occurring in the cortex and bundles of fibers arranged in fascicular position above the vascular bundles; secondary xylem dominated by fibers, vessels dispersed, reticulate, mainly strongly bordered, up to 79 µm in diameter; medullary rays bi- and multiseriate; pith missing; crystalloids, laticifers missing; secretory ducts beside endoSDs missing;

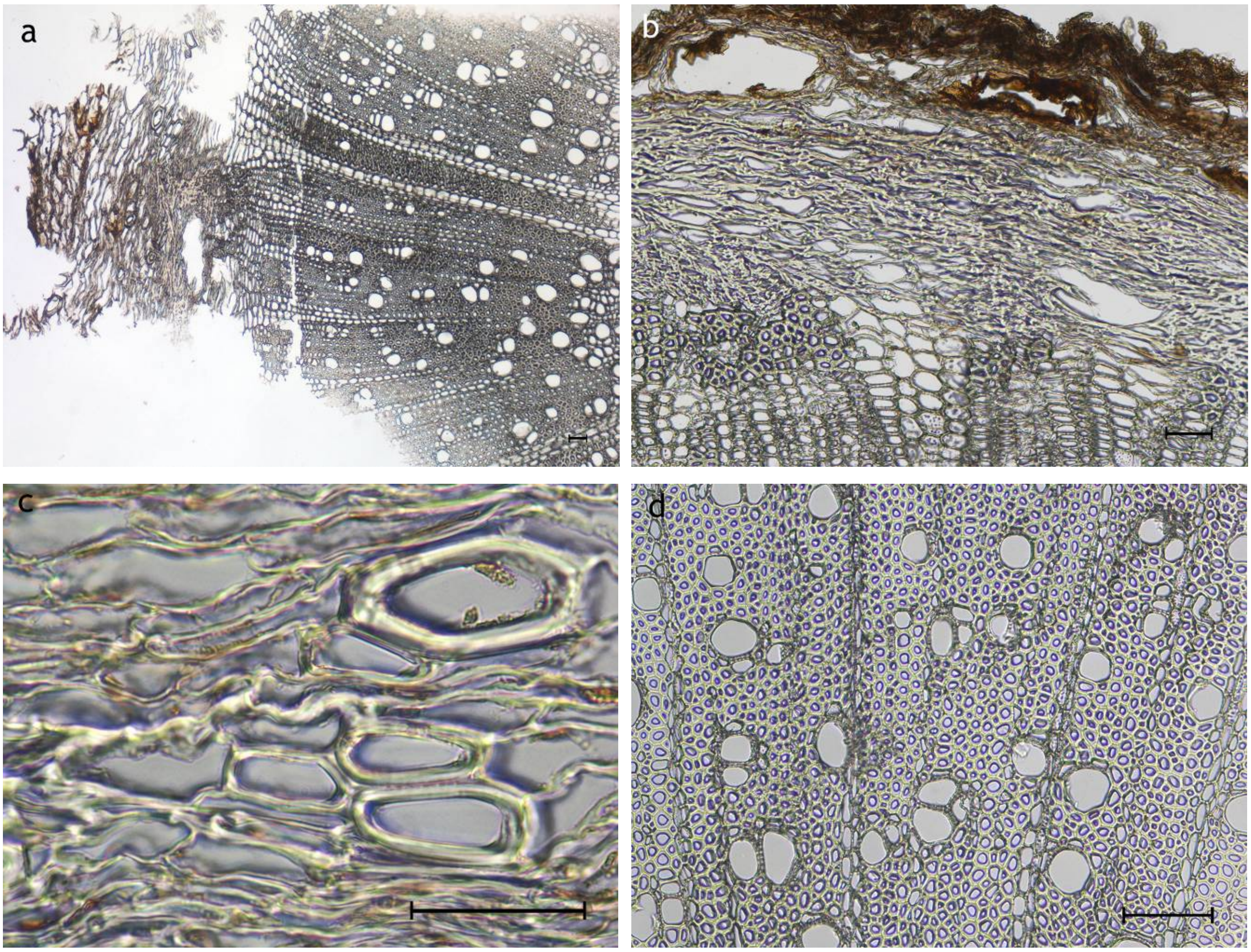

Cirsium vulgare root: a: Overview showing the extension and arrangement of tissues: narrow cortex enduring, secondary phloem broad, but of lesser radial extension than the vascular cylinder; bark / suberized cortex and secondary phloem with sclereids, vascular cylinder with bi- and multiseriate medullary rays; b: cortex with endoSDs, secondary phloem with fibers arranged in bundles; c: sclereids of secondary phloem; d: xylem dominated by fibers and with dispersed vessels, biseriate medullary rays in regular arrangement; a–d: transverse sections;

6. *Silybum marianum*

secondary root: phellem thin-walled; cortex lost in course of rhytidome formation; endoSDs lost together with the cortex; small secondary phloem usually broader than cortex, but of lesser radial extension than the vascular cylinder, with fibers single and arranged in bundles in fascicular position above the vascular cylinder between the phloem rays; secondary xylem with mainly parenchymatous cells (often slightly thickened walls), few fibers; vessels in groups, strongly bordered, up to 136 µm in diameter, short in longitudinal section; medullary rays usually up to 5 cells in a row, very regularly arranged; pith missing; crystalloids, sclereids missing; secretory ducts beside endoSDs missing;

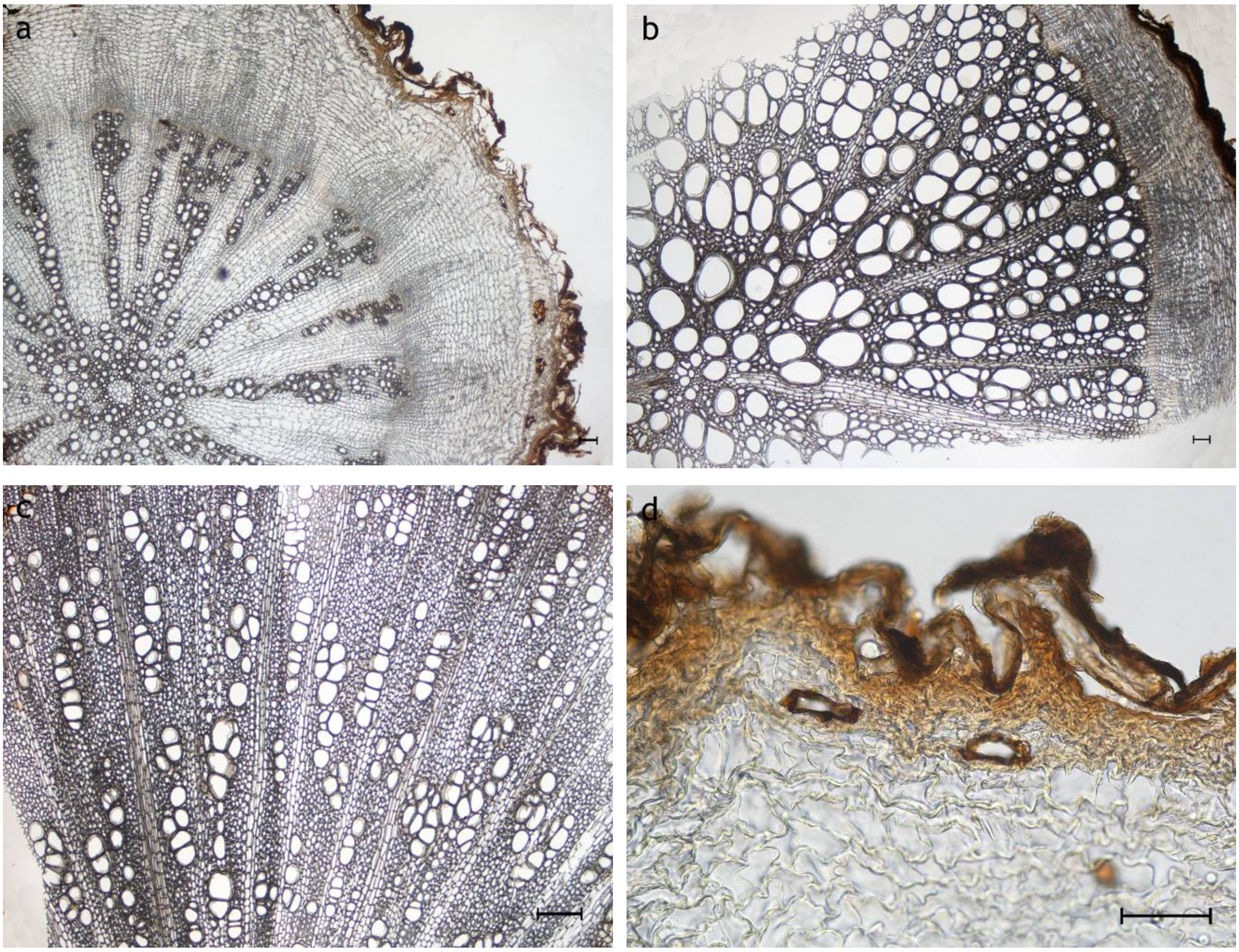

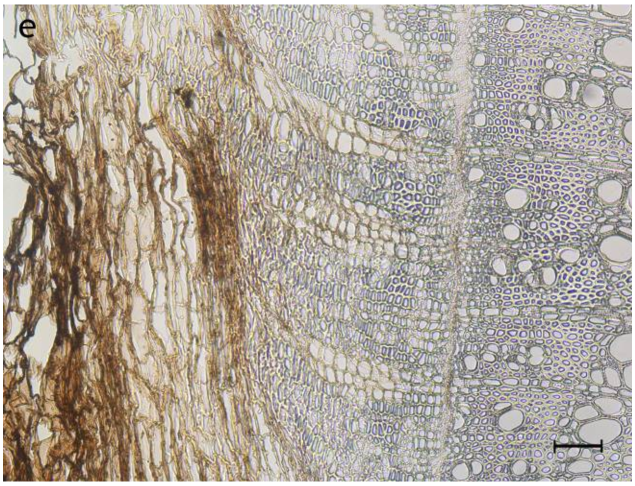

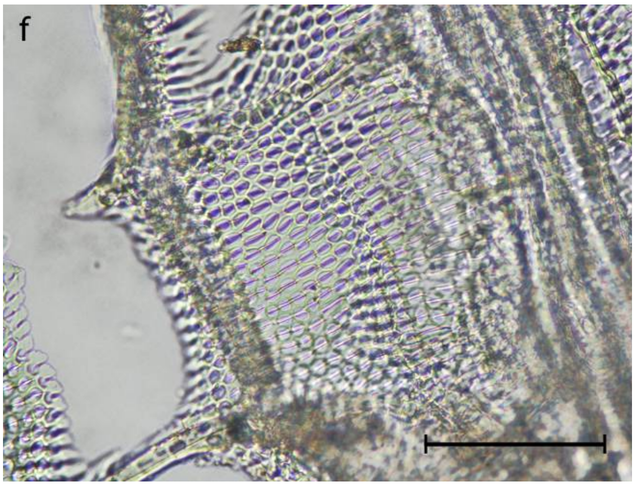

Silybum marianum root: a: Young root (cultivated plant): overview showing the extension and arrangement of tissues: small cortex with endoSDs, broad sec. phloem, but of lesser radial extension than the vascular cylindervascular cylinder with multiseriate medullary rays, vessels in rows; b: older root: overview showing the extension and arrangement of tissues: cortex lost, sec. phloem broader than cortex but of far lesser radial extension than the vascular cylinder; c: xylem dominated by fibers, vessels more or less in groups, medullary rays up to 5 cells in a row in regular arrangement; d: cortex with endoSDs; e: suberized cortex and sec. phloem with fibers arranged in bundles in fascicular position above the vascular cylinder between the phloem rays; f: short, strongly bordered vessels; a–e: transverse sections; f: longitudinal section;

7. *Centaurea jacea*

Root:

secondary root: rhizodermis brown; cortex enduring, with distinct sclereids arranged in groups surrounded by parenchyma cells: shape roundish to elliptic in transverse view, in longitudinal view resembling the adjacent parenchyma cells in size and shape, thick-walled, strongly lignified, with simple pits; distinct endodermis with endoSDs – usually up to 6-7 surrounding cells at maximum; secondary phloem with sclereids of the same type occurring in the cortex, with rarels scattered fibers; broad vascular cylinder dominating in extension with prim. xylem visible; secondary xylem dominated by fibers, few vessels dispersed over the transverse section, reticulate, simple or weakly bordered, up to 57 µm in diameter; medullary rays varying from narrow to broad rays, can even appear almost raylessness in transverse section because of thickened cell walls of ray cells; secretory ducts of type SD4 (secretion by parenchyma cells / sclereidal cells into ordinary intercellular spaces) always associated with sclereids occurring in central position of the pith as well as in cortex and secondary phloem; pith of unlignified, slightly thickened parenchymous cells with just few pits visible in transverse section, with sclereids; cristalloids, laticifers missing;

Rhizome:

rhizome with secondary growth: rhizodermis brown; small cortex enduring but tissue frequently ruptured / obliterated, with sclereids single or in groups mainly in conjunction with SD4 (see above); endoSDs lost or crushed together with rupturing of the cortex; secondary phloem usually broader than cortex, but of lesser radial extension than the vascular cylinder broad, with sclereids of the same type occurring in the cortex and fibers arranged in bundles which may build caps over vascular bundles; secondary xylem with few vessels usually in groups cumulative to the center, reticulate, simple or weakly bordered, up to 45 µm in diameter; medullary rays with partly thickened cell walls; secretory ducts type SD2a possible within secondary phloem, type SD4 in conjunction with sclereids in cortex, secondary phloem fascicular and interfascicular and pith possible; pith of cells with slightly thickened, pitted cell wall, with sclereids (as mentioned above); crystalloids, laticifers missing;

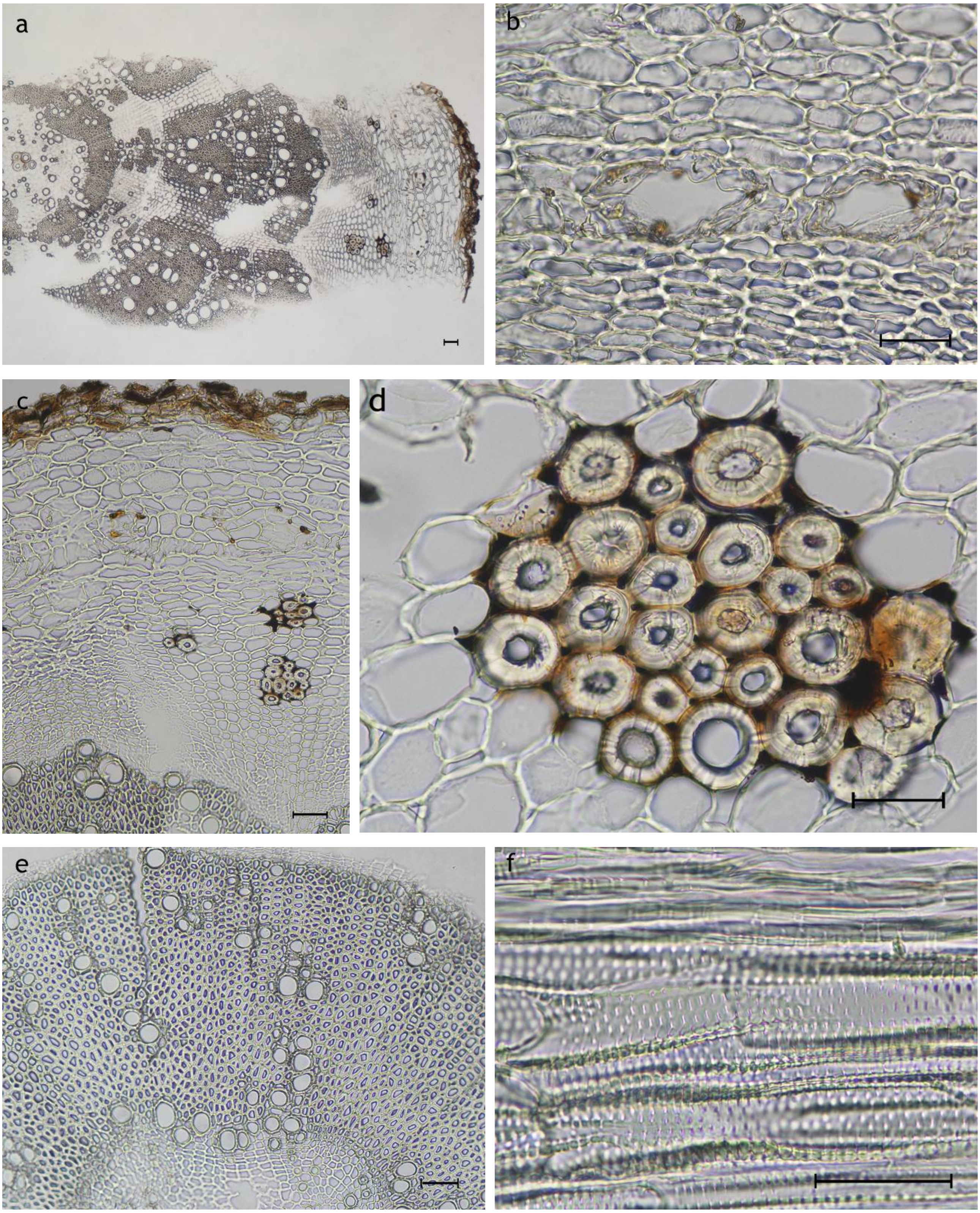

a
b
c
d
e
f

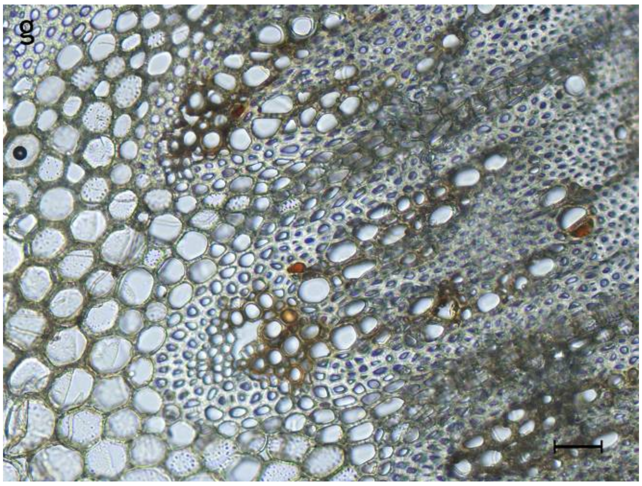

Centaurea jacea root: a: Overview showing the extension and arrangement of tissues: the secondary xylem is the most expanded component; b: endoSDs of sec. phloem; c: overview showing sclereids in conjunction with SD4 within the secondary phloem, endodermis with endoSDs, cortex and rhizodermis; d: sclereids positioned in the pith associated with secretory ducts type SD4; e: secondary xylem dominated by fibers, with few vessels dispersed; f: pitted vessels; rhizome: g: transition between pith and vascular bundles; a–e,g,h: transverse sections; f: longitudinal sections

8. *Centaurea scabiosa*

secondary root: phellem thin-walled, sometimes with crystalloids; cortex durable or lost in course of rhytidome formation, with occasionally sclereids single or in groups resembling the adjacent parenchyma cells in size and shape; distinct endodermis with endoSDs lost together with cortex; secondary phloem dominant, comparable in extension to the vascular cylinder, with sclereids of the same type occurring in the cortex and pitted fibers single or in bundles; secondary xylem with fibers in tangential bands more or less alternating with bands of parenchymatous cells, vessels circularly arranged relative to the centre of the xylem, reticulate and strongly bordered, up to 114 µm in diameter; medullary rays broad, multiseriate, unlignified; secretory ducts type SD3 (lysigenous development, large inner diameter – can fill the whole space from one phloem ray to the next) located in fascicular position between the phloem rays within the secondary phloem; secretory ducts type SD1 and SD2 forming a triangular pattern around SD3 – fascicular positioned; pith missing; laticifers missing;

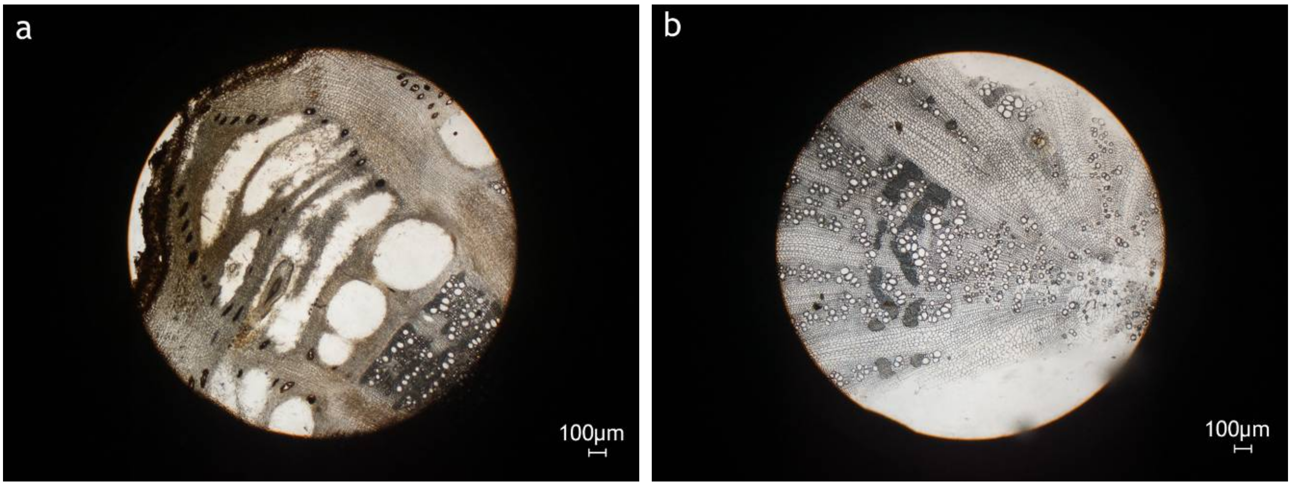

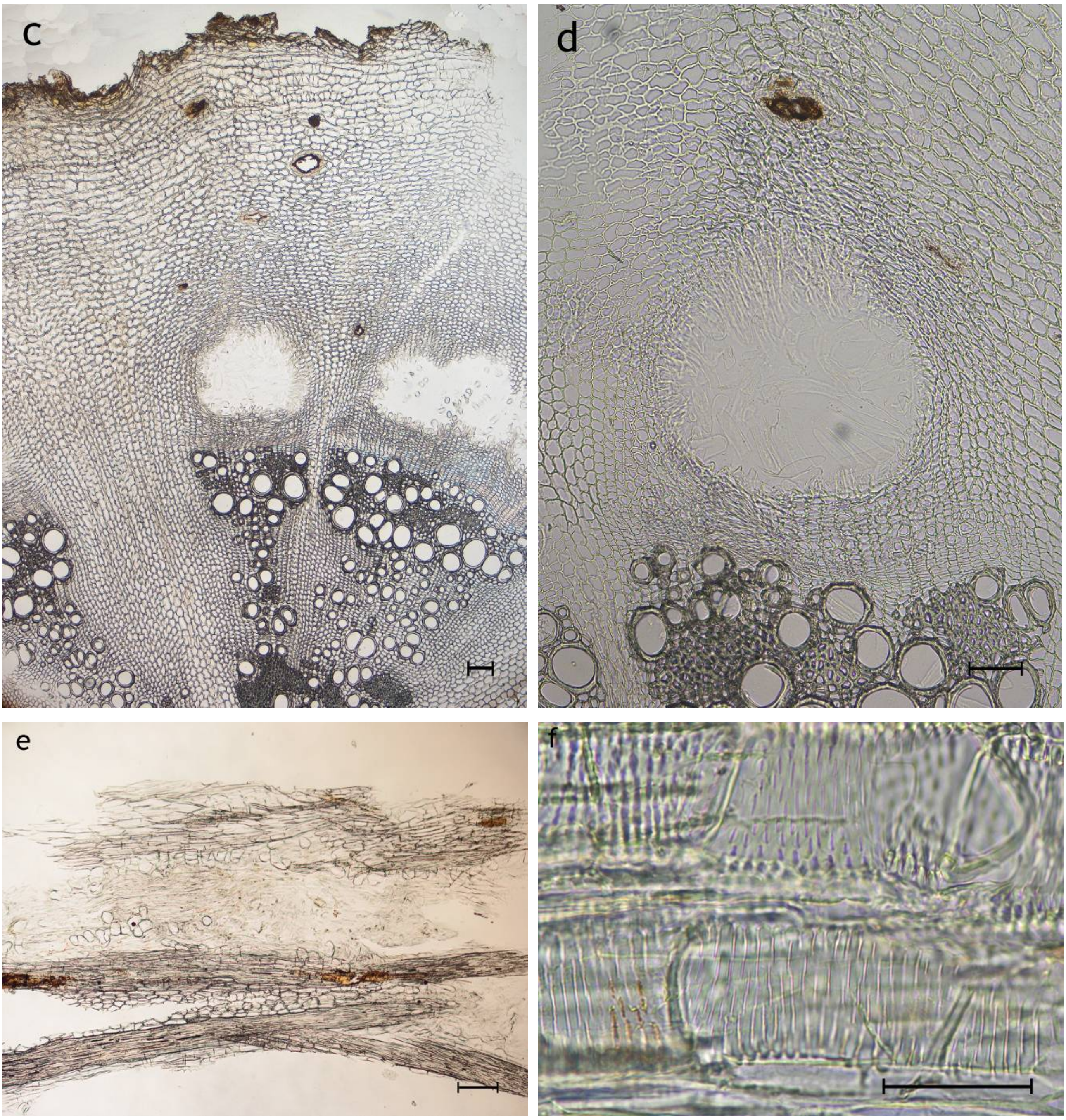

Centaurea scabiosa root: a: Overview showing the extension of the secondary phloem with secretory ducts SD1 -2 / SD3; SD1-2 form a triangular pattern around SD3; b: secondary xylem with fibers in tangential bands more or less alternating with bands of parenchymatous cells; c: cortex with distinct endodermis and endodermal resin duct, secretory ducts SD3 located in fascicular position between the phloem rays; d: secretory duct type SD3 (lysigenous development) with SD2 at the top; e: secretory duct type SD3; f: reticulate vessels; a–d: transverse sections; e,f: longitudinal sections;

9. *Centaurea cyanus*

secondary root: phellem narrow, thin-walled, sometimes with crystalloids; cortex enduring, possibly with sclerenchymatous thickening of cells though distinct sclereids are missing; distinct endodermis with endoSDs – usually up to 10 surrounding cells at maximum; secondary phloem narrow, in younger roots almost invisible; vascular cylinder dominating;; secondary xylem dominated by fibers, few vessels either dispersed over the transverse section or arranged in tangential bands, reticulate, mainly strongly bordered, up to 82µm in diameter; medullary rays up to 5 cells in one row, can even appear almost raylessness in transverse section because of thickening of cell walls of ray cells; secretory ducts type SD2b in fascicular position within secondary phloem; pith missing; sclereids, laticifers missing; fibers in sec. phloem missing;

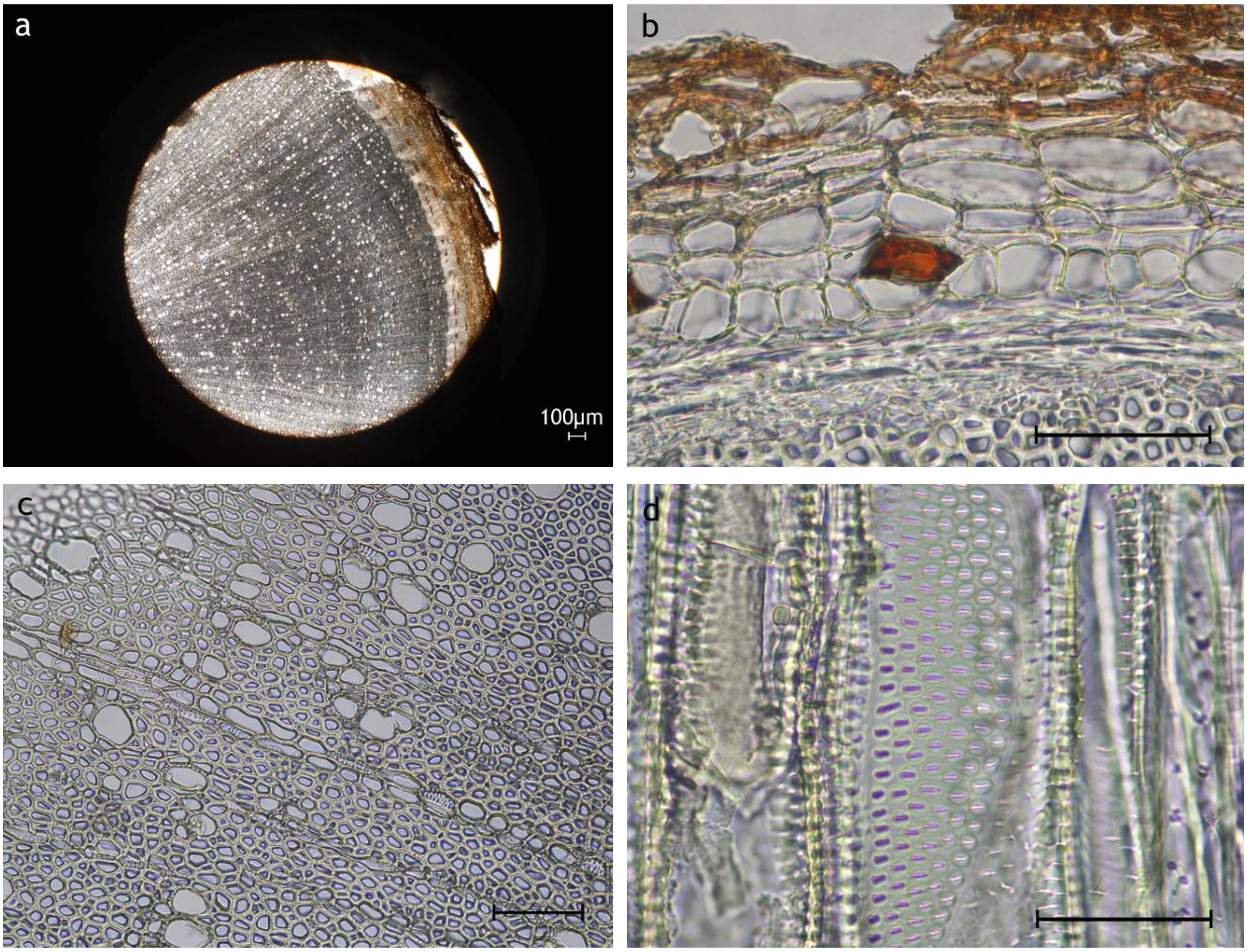

Cyanus cyanus root: a: Overview showing the extension and arrangement of tissues: vascular cylinder dominating, small secondary phloem, cortex partly suberized, endoSDs enduring; b: endodermal resin duct; c: xylem dominated by fibers, medullary rays mono- and biseriate; d: strongly bordered vessels; a–c: transverse sections; d: longitudinal section;

10. *Cnicus benedictus*

secondary root: phellem narrow, thin-walled, sometimes with crystalloids; cortex enduring; endoSDs with small lumen; small secondary phloem usually broader than cortex, but of lesser radial extension than the vascular cylinder, with fibers arranged in bundles in fascicular position above the vascular cylinder between phloem rays; secondary xylem with fibers, vessels either more or less circularly arranged relative to the centre of the xylem or dispersed, strongly bordered, up to 93µm in diameter; medullary rays usually narrow, bi- or multiseriate, regularly arranged; pith missing; sclereids, laticifers missing; secretory ducts beside endoSDs missing;

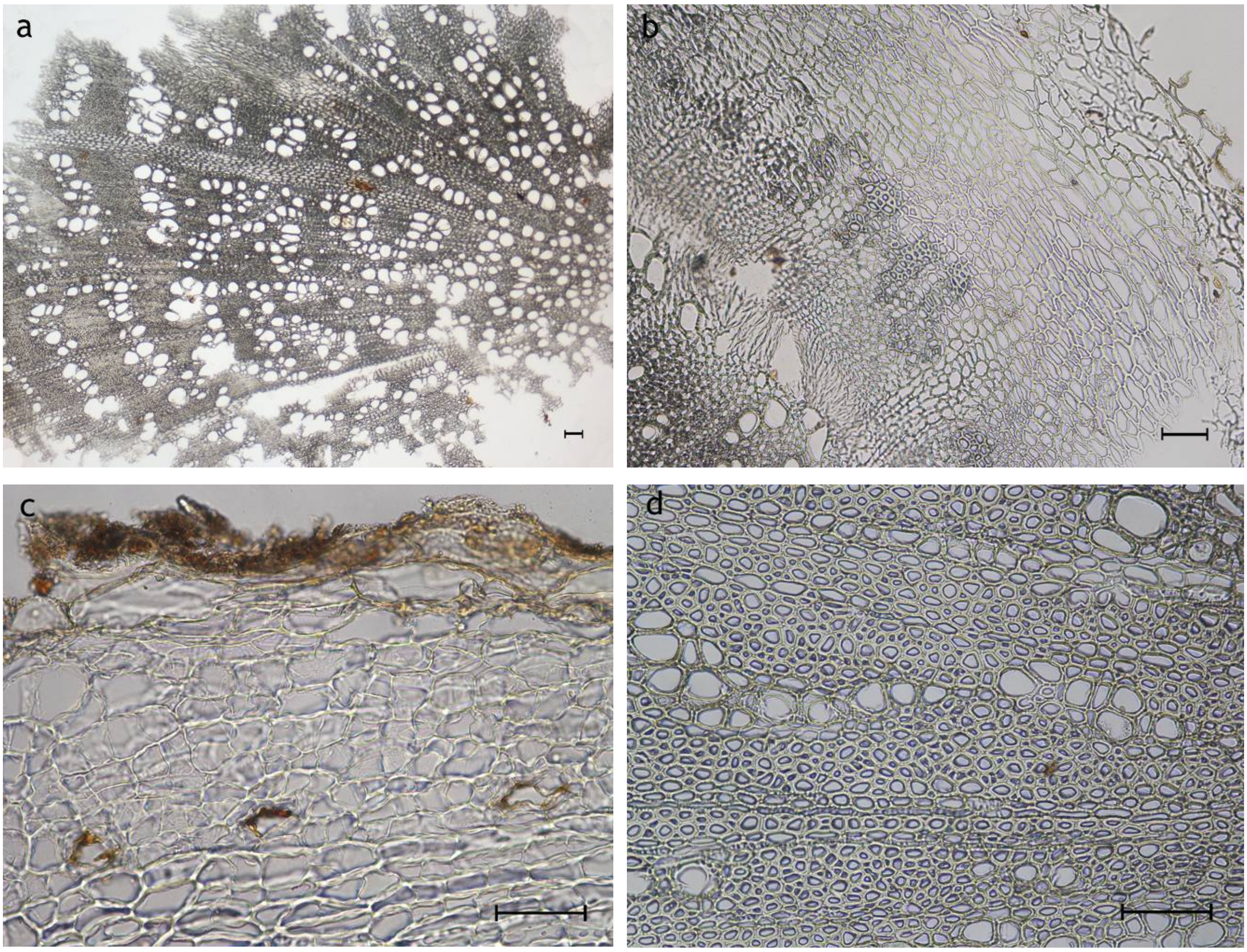

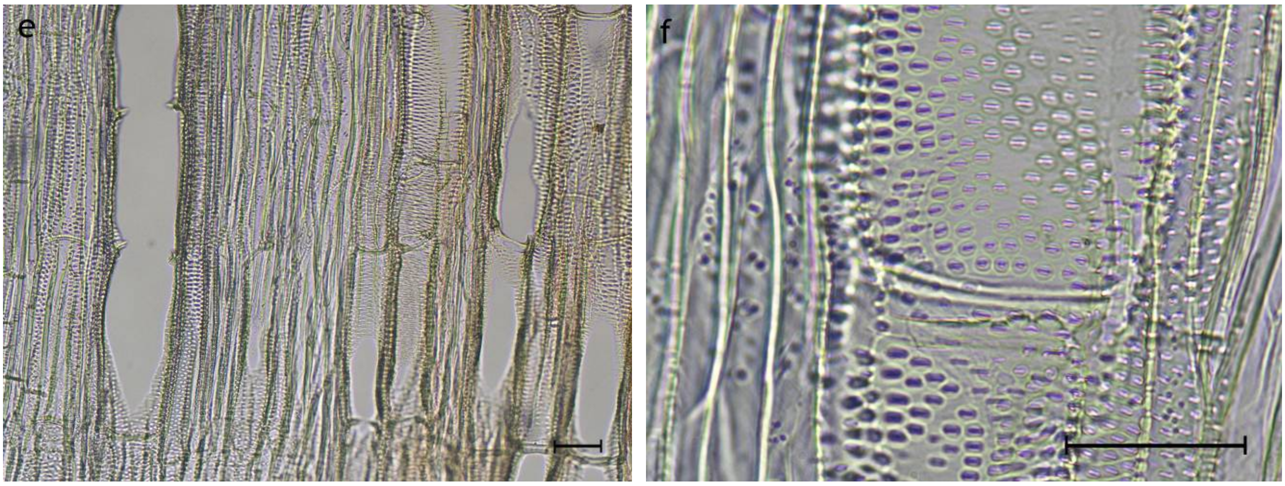

Cnicus benedictus root: a: Overview showing the extension and arrangement of tissues: small cortex with endoSDs, broad secondary phloem, but of lesser radial extension than the vascular cylinder cortex, vascular cylinder; b: overview showing the anatomy of the secondary phloem; c: endodermis with regularly arranged endoSDs with small lumina; d: xylem dominated by fibers, medullary rays mono- and biseriate; e: overview of secondary xylem; f: strongly bordered vessels; a–d: transverse sections; e,f: longitudinal section;
